# LLM‐Assisted Design of Ultralow‐κ Chalcogenides

**DOI:** 10.1002/advs.78175

**Published:** 2026-10-15

**Authors:** Yujie Liu, Congran Jia, Yuzhou Hao, Xuejie Li, Yang Wang, Artem Oganov, Jun Sun, Xiangdong Ding, Turab Lookman, Zhibin Gao

**Affiliations:** ^1^ State Key Laboratory for Mechanical Behavior of Materials State Key Laboratory of Porous Metal Materials School of Materials Science and Engineering Xi'an Jiaotong University Xi'an China; ^2^ Skolkovo Institute of Science and Technology Moscow Russia; ^3^ AiMaterials Research LLC Santa Fe New Mexico United States

**Keywords:** large language models, lattice thermal conductivity, llm‐assisted materials search, thermoelectrics, universal interatomic potentials

## Abstract

Accelerating the discovery of materials with extreme lattice thermal conductivity (κ) is limited by the high cost of first‐principles screening. Here, we develop an LLM‐assisted low‐κ discovery workflow, where Bayesian optimization (BO) proposes candidates, an LLM performs evidence‐conditioned re‐ranking, and physics‐based structure generation, stability validation, and κ calculations serve as the acceptance criteria. This integration enables efficient candidate prioritization while maintaining rigorous physical validation. The workflow identifies dynamically stable Ag‐based chalcogenides, including Ag_3_SbSe_4_, Ag_4_TiSe_4_, and Ag_4_TiTe_4_. Mechanistic analysis reveals that their ultralow κ originates from strong phonon renormalization and coherence‐dominated heat transport. Ag_3_SbSe_4_ is predicted to reach a maximum ZT of 2.94 at 500 K due to its ultralow κ of 0.11 W $m^{-1}$ $K^{-1}$. Furthermore, automated high‐throughput screening of ternary compounds containing 14 elements identifies 2742 dynamically stable materials with κ≤1.0 W $m^{-1}$ $K^{-1}$. These results demonstrate that evidence‐conditioned LLM re‐ranking effectively enhances BO‐driven, physics‐validated materials discovery.

## Graphical abstract

An AI assisted, physics guided workflow accelerates the discovery of ultralow thermal conductivity materials by combining Bayesian optimization, evidence conditioned LLM reasoning, and automated physics‐based validation. The framework uncovers dynamically stable Ag based chalcogenides with ultralow thermal conductivity and promising thermoelectric performance, enabling efficient exploration of previously unexplored regions of low thermal conductivity materials space.

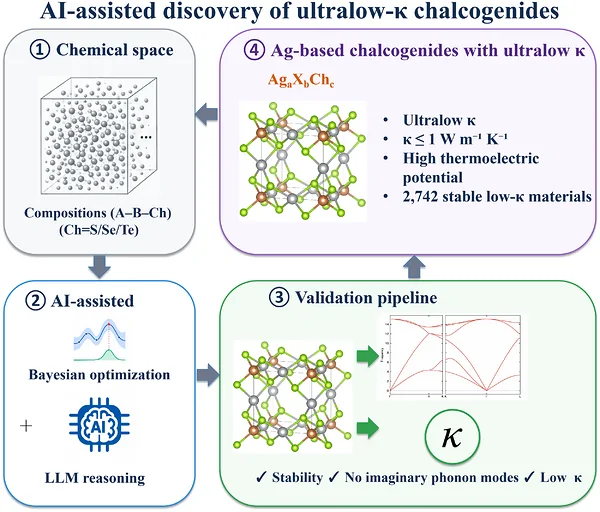

## 1 Introduction

Lattice thermal conductivity (κ) serves as a decisive descriptor for thermal transport in nonmetallic crystals, underpinning key technologies from sustainable thermoelectric energy conversion to heat dissipation in high‐power electronics [1, 2, 3, 4, 5]. However, accelerating the discovery of materials with extreme κ values remains a persistent bottleneck. Although first‐principles approaches, solving the phonon Boltzmann transport equation (BTE) provide high‐fidelity predictions [6, 7, 8], their steep computational cost renders high‐throughput screening of complex, low‐symmetry crystals unfeasible [9, 10]. Similarly, classical molecular dynamics (MD) is often compromised by the scarcity of generalizable and accurate interatomic potentials [11, 12, 13]. When compounded by the resource‐intensive nature of experimental synthesis, these limitations create a significant barrier to exploring the vast chemical space for next‐generation thermal materials.

A transformative shift toward knowledge‐driven computational workflows, powered by generative artificial intelligence and foundation models, is currently reshaping the materials discovery paradigm. Pioneering exploration efforts exemplified by GNoME have drastically expanded the known inorganic material space through active learning [14, 15]. A cornerstone of this evolution is the development of Universal Interatomic Potentials (UIPs) that act as general‐purpose foundation models across the periodic table. While seminal models such as M3GNet [16] and CHGNet [17] established the feasibility of universal property prediction, cutting‐edge architectures like MACE [18] and SevenNet [19] now provide superior equivariance and physical consistency.

Crucially, recent evaluations of phonon spectra and thermodynamic properties demonstrate that MatterSim [20] has achieved state‐of‐the‐art accuracy, significantly reducing errors in maximum phonon frequencies and heat capacities relative to predecessors. Alongside these predictive advances, the emergence of generative models, such as the Crystal Diffusion Variational Autoencoder (CDVAE) [21] and DiffCSP [22], has enabled the de novo design of crystal structures with specific symmetry constraints. More recently, advances including CrystaLLM [23] and MatterGen [24] have further extended these capabilities by treating crystal generation as a multi‐property optimization task or a sequence modeling question, unlocking the discovery of stable structures previously inaccessible to traditional databases.

However, despite the generative prowess of these models and the efficiency of universal potentials, purely data‐driven approaches often struggle to ensure experimental realizability due to a lack of intrinsic physical reasoning. To bridge this critical gap, LLMs are increasingly being integrated into tool‐augmented scientific workflows [25], empowering the discovery process with knowledge‐driven reasoning. Pioneering systems such as Coscientist [26] and ChemCrow [27] have showcased the potential to assist with planning and executing complex chemical tasks by augmenting LLMs with domain‐expert tools and literature access. Building upon this foundation, advanced frameworks like LLaMP [28] and MAPPS [29] further refine this paradigm by establishing hierarchical reasoning structures. By seamlessly bridging high‐level strategic planning with low‐level physics‐based simulations, these tool‐augmented workflows effectively mitigate the risk of hallucination and enforce theoretical consistency [30].

Here, we present an LLM‐assisted design framework that orchestrates LLM‐assisted candidate prioritization with high‐throughput, physics‐guided validation to accelerate the identification of materials with ultralow κ. Here, “LLM‐assisted design” denotes workflow‐level candidate prioritization: Bayesian optimization (BO) generates candidates; the LLM only re‐ranks a subset; physics validation determines final acceptance. “Physics‐guided” refers to the workflow‐level use of physical constraints, embedded κ models, and DFT validation criteria. Our closed‐loop active learning system integrates four synergistic modules (Figure 1). The workflow begins with Gaussian Process Regression (GPR [31])‐based surrogate modeling of the composition–κ landscape and Bayesian optimization, where candidates are proposed by a Metropolis–Hastings Markov chain Monte Carlo sampler (MCMC) [32] and ranked by the Expected Improvement (EI) criterion. The BO stage provides a quantitative acquisition ranking using composition‐only features, whereas the LLM module is used only for qualitative re‐ranking of the top 20 BO‐ranked compositions based on mechanism‐level evidence. The LLM operates exclusively on the top 20 BO‐ranked candidates and does not participate in global search. The top 10 compositions after this re‐ranking are passed to structure generation and deterministic validation. The top 20 BO‐ranked compositions are then processed by the LLM‐assisted evidence‐conditioning module, which integrates the internal theory document and controlled public web‐search evidence enrichment through Agno WebSearchTools [33] to support qualitative re‐ranking. The selected compositions are then processed by an automated structure generation and calculation pipeline, where validation is performed by structural relaxation, phonon calculations, and κ evaluation rather than by the LLM. In this pipeline, the top 10 compositions selected after qualitative LLM re‐ranking are structurally instantiated by CrystaLLM, which generates five candidate crystal structures for each selected composition. Subsequently, these structures are rigorously verified for dynamical stability using the state‐of‐the‐art universal potential, MatterSim. Finally, κ is rapidly assessed via the KappaP component implemented in this workflow for rapid κ screening.

**FIGURE 1:**
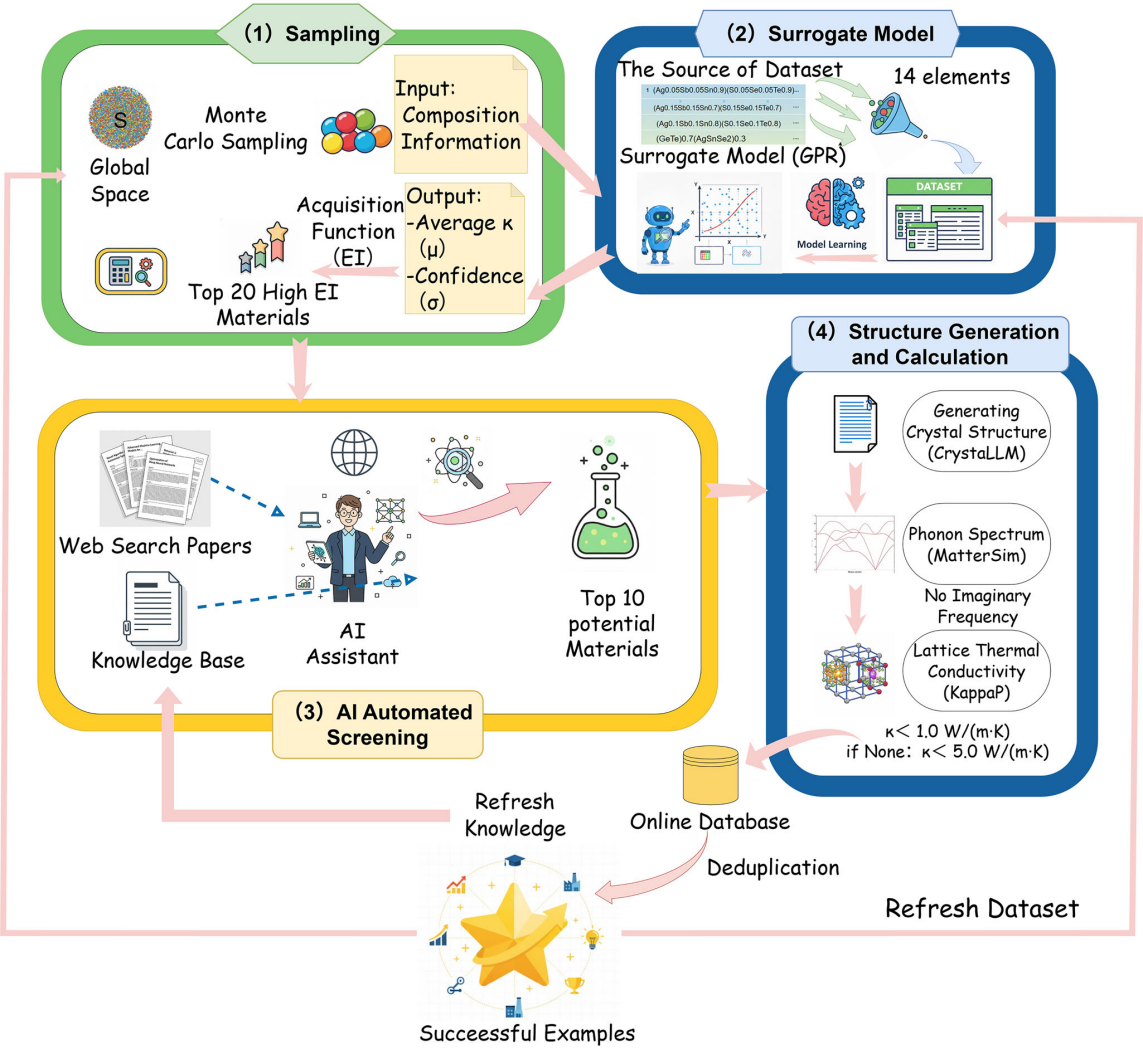
Schematic illustration of the closed‐loop active learning framework for LLM‐assisted prioritization and physics‐validated discovery of ultralow‐κ materials. (1) **Markov chain Monte Carlo (MCMC) sampling in discrete composition space**: Candidate compositions are generated using a Metropolis–Hastings sampler over ternary formulas with a maximum atom count. Proposal moves include element substitution, stoichiometric ±1 perturbations, and ternary switches under atom‐count constraints. Acceptance is governed by a score balancing low predicted κ, model uncertainty, and EI‐driven improvement. When historical successful or dynamically stable cases exist, the chain is initialized from a randomly selected seed composition; otherwise, it starts from a randomly generated ternary composition. (2) **Bayesian optimization**: A Gaussian process regression (GPR) surrogate is trained on the evolving dataset (initialized from the provided dataset). Expected Improvement (EI) guides acquisition, and the MCMC samples are scored and ranked to select high‐value candidates for downstream evaluation. (3) **LLM‐assisted evidence‐conditioned re‐ranking**: The LLM module re‐ranks the top 20 BO‐ranked candidates using the internal theory document and controlled public web‐search evidence enrichment through Agno WebSearchTools, retaining the top 10 compositions for structure generation. (4) **Generative Validation Pipeline**: CrystaLLM generates five candidate structures for each selected composition, dynamically relaxed and verified for dynamical stability via MatterSim, and assessed for κ using the KappaP framework. Closing the Loop: Validated data are fed back to refine the GPR surrogate and update the evidence records used in subsequent re‐ranking cycles.

The KappaP framework integrates a pre‐trained crystal graph convolutional neural network (CGCNN) with the physics‐based Slack model (Method Section 4.6) (Figure 2). It first predicts the bulk and shear moduli, which are then used to derive the Grüneisen parameter and acoustic Debye temperature. These derived parameters enable the estimation of κ with high fidelity. Validated discoveries are fed back to refine the statistical surrogate and the structured evidence records used by the LLM‐assisted re‐ranking module.

**FIGURE 2:**
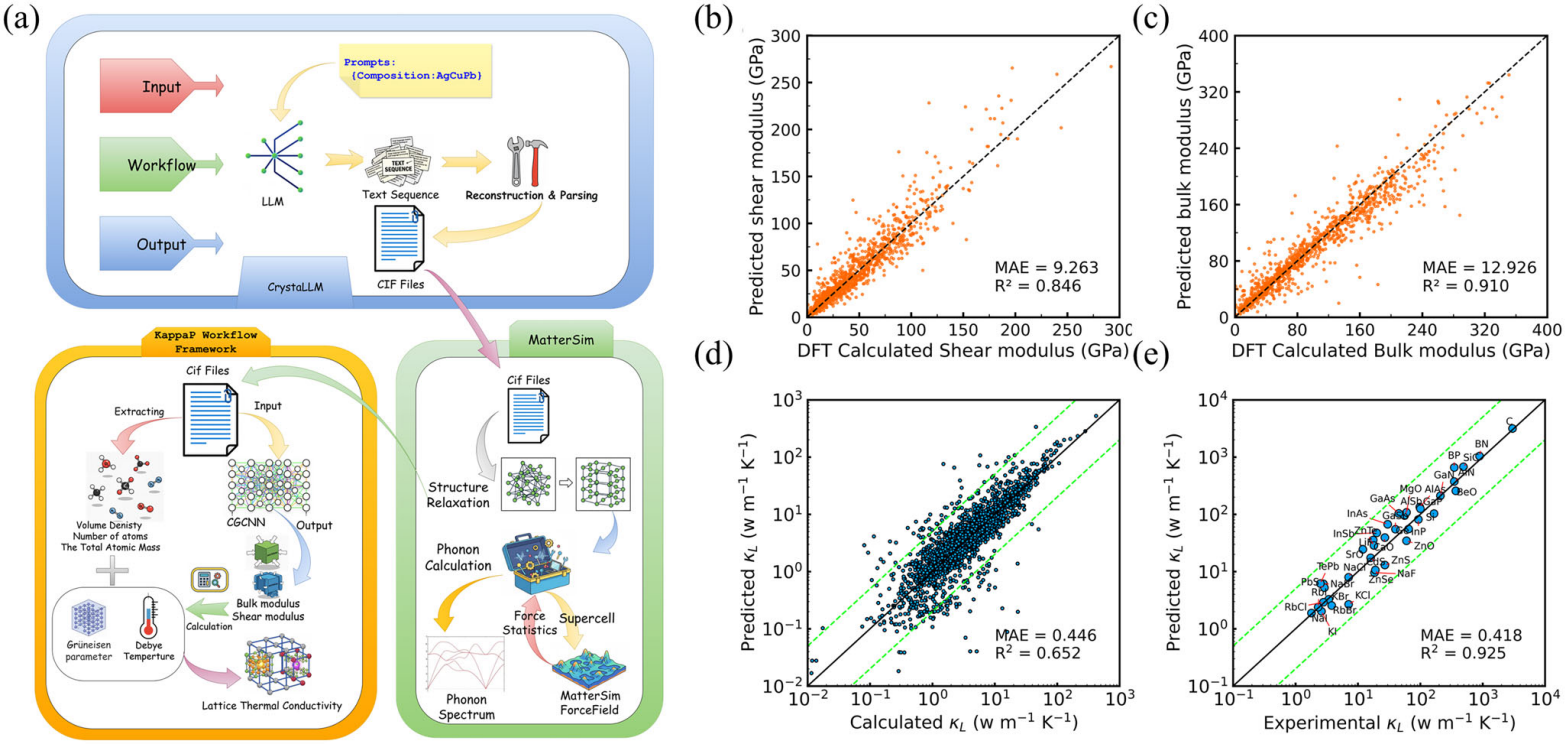
Architecture and rigorous validation of the high‐throughput computational pipeline. (a) Schematic of the integrated validation workflow. The pipeline orchestrates three advanced deep learning modules: (1) CrystaLLM (blue) generates 3D crystal structures from chemical compositions via LLM‐assisted decoding; (2) MatterSim (green) performs high‐fidelity structural relaxation and phonon calculations using universal potentials to verify dynamical stability; and (3) Kappa Predictor (KappaP, orange) estimates κ by predicting elastic moduli via a Crystal Graph Convolutional Neural Network (CGCNN) and integrating them with Grüneisen parameters into the Slack model. (b, c) Validation of elastic moduli. Parity plots comparing CGCNN predictions against DFT‐calculated ground truth for (b) shear modulus (G) and (c) bulk modulus (B) on the testing dataset, demonstrating high predictive fidelity. (d, e) Dual‐level benchmarking of κ predictions. Comparison of KappaP estimates against (d) 2535 theoretical entries from the AFLOW database and (e) 38 experimental measurements at 300 K [34, 35]. Dashed lines delineate a half‐order‐of‐magnitude deviation margin, confirming the model's reliability across both theoretical and experimental domains.

As a proof‐of‐concept application, we deployed this closed‐loop active learning system to explore an A–B–Ch ternary chalcogenide composition family constructed from 14 low‐κ‐relevant elements, with ≤ 10 atoms per conventional cell, where A denotes Ag, Cu, In, Sn, or Pb, B denotes As, Sb, Ge, Bi, Ti, or V, and Ch denotes S, Se, or Te. Through the automated structure generation and calculation pipeline, we successfully generated and screened 2742 dynamically stable materials with κ below 1 W $m^{-1}$ $K^{-1}$. The workflow identified several previously unexplored, dynamically stable Ag‐based chalcogenide candidates distinguished by ultralow κ and promising downstream thermoelectric potential. Downstream electronic‐transport calculations indicate predicted ZT values of 2.94 at 500 K for Ag_3_SbSe_4_, 2.47 at 700 K for Ag_4_TiSe_4_, and 3.68 at 700 K for Ag_4_TiTe_4_, but these values are not used as the BO or LLM screening objective. These results support a constrained LLM‐assisted workflow in which evidence‐conditioned re‐ranking reduces the number of candidates passed to expensive validation while final materials acceptance remains governed by deterministic physics calculations.

## 2 Results

To rapidly discover materials with low κ, we established a closed‐loop active learning framework (Figure 1) that seamlessly integratesBO, LLM‐assisted reasoning, and a unified high‐throughput validation pipeline. The cycle is initialized using a GPR surrogate model, trained on a curated seed dataset of 220 low‐κ compounds encompassing 14 representative elements. The use of GPR is motivated by its probabilistic uncertainty estimates, which are required for EI acquisition. As discussed in Section 4.2 and Figure S3, the composition‐only representation gives the best cross‐validation performance among the tested feature sets, outperforming both the selected elemental descriptors and the combined composition‐plus‐elemental‐descriptor representation. Navigation of the chemical space is driven by the EI acquisition function, employing an adaptive hybrid sampling strategy that balances 70% acquisition‐guided optimization with 30% random exploration. At each iteration, BO first ranks candidate compositions by the acquisition function. The top 20 BO‐ranked compositions are then qualitatively re‐ranked by the LLM module using mechanism‐level evidence from the internal theory document and public web‐search summaries, including lone‐pair effects and lattice anharmonicity. The top 10 compositions after this re‐ranking are submitted to structure generation and deterministic validation. Upon selection, the prioritized candidates are channeled into a rigorous high‐throughput computational validation pipeline (Figure 2a), which synergistically deploys three advanced deep learning modules. First, CrystaLLM instantiates composition sequences into plausible three‐dimensional crystal structures by decoding learned crystallographic patterns [23]. These generated structures then undergo high‐fidelity geometric relaxation and phonon spectrum calculations via MatterSim [36], acting as a critical filter to purge dynamically unstable candidates characterized by imaginary phonon frequencies. Finally, the dynamically stable structures are characterized by the KappaP framework, which integrates pre‐trained CGCNN models with the physics‐based Slack model. By directly predicting bulk and shear moduli from the crystal structure, KappaP combines these elastic constants with derived Grüneisen parameters and acoustic Debye temperatures to yield instantaneous, physics‐guided estimates of κ.

In this stage, CrystaLLM generates five candidate structures for each of the 10 selected compositions before relaxation and stability filtering. Ensuring the predictive fidelity of the KappaP is paramount for the integrity of the discovery pipeline. As demonstrated by validating against Density Functional Theory (DFT) calculations (Figure 2b,c), the neural network backbone predicts elastic moduli with high precision, achieving coefficients of determination ($R^{2}$) approaching 0.9. Beyond elastic parameters, we implemented a stringent dual‐validation strategy for the final κ estimates, spanning both theoretical and experimental domains. A large‐scale comparison against 2,535 cubic materials from the AFLOW database (Figure 2d) reveals that 95.15% of predictions fall within a half‐order‐of‐magnitude margin, indicating excellent generalizability. Most critically, benchmarking against experimental measurements for 38 diverse materials (Figure 2e and Table. 1) yields a low Mean Absolute Error (MAE) of 0.418 W $m^{-1}$ $K^{-1}$ and a remarkable $R^{2}$ of 0.925. This good alignment with experimental reality cements the framework as a robust and reliable filtering tool for high‐throughput screening.

**TABLE 1:** Predicted and experimental room‐temperature κ values for compounds from the literature. The CIFs for these materials were obtained from the Materials Project. Here, $\theta_{a}$ represents the acoustic Debye temperature calculated from Equation (7); n denotes the number of atoms in the primitive cell; γ is the experimental Grüneisen parameter; V is the volume of the primitive cell; M is the total atomic mass; $\kappa_{code}$ is the calculated κ derived from Equation (1); and $\kappa_{exp}$ denotes the experimentally measured values.

Materials	ID‐number	n	$\theta_{a}$ (K)	γ	V (Å 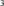 )	M (amu)	$\kappa_{code}$ (W $m^{-1}$ $K^{-1}$)	$\kappa_{exp}$ (W $m^{-1}$ $K^{-1}$)
C [34]	mp‐66	2	1800.252	0.750	11.410	24.022	3155.309	3000
Si [34]	mp‐149	2	473.641	1.060	40.888	56.172	103.223	166
Ge [34]	mp‐32	2	275.718	1.060	47.847	145.280	55.496	65
AlAs [34]	mp‐2172	2	307.694	0.660	47.126	101.904	133.884	98
AlSb [34]	mp‐2624	2	224.333	0.600	60.561	148.742	95.520	56
BN [35]	mp‐1639	2	1433.102	0.954(By Equation (7))	11.919	24.818	1042.968	890
BP [34]	mp‐1479	2	936.611	0.922(By Equation (7))	23.500	41.785	658.408	350
CaO [34]	mp‐2605	2	474.978	1.570	28.332	56.077	39.340	27
GaAs [34]	mp‐2534	2	270.511	0.750	47.532	144.645	103.718	45
GaP [34]	mp‐2490	2	330.097	0.750	41.737	100.697	125.636	100
GaSb [34]	mp‐1156	2	194.368	0.750	60.133	191.483	55.086	40
InAs [34]	mp‐20305	2	180.058	0.570	59.050	189.740	67.207	30
InP [34]	mp‐20351	2	217.606	0.600	52.840	145.792	81.656	93
InSb [34]	mp‐20012	2	144.394	0.560	72.965	236.578	47.500	20
KBr [34]	mp‐570891	2	138.860	1.450	66.111	119.002	3.292	3.4
KCl [34]	mp‐23193	2	151.828	1.450	65.033	74.551	2.681	7.1
KI [34]	mp‐22898	2	101.745	1.450	92.743	166.002	2.022	2.6
LiF [34]	mp‐1009009	2	609.150	1.500	16.768	25.939	35.605	17.6
MgO [34]	mp‐1265	2	728.881	1.440	19.279	40.304	108.546	60
NaBr [34]	mp‐22916	2	187.587	1.500	54.749	102.894	6.119	2.8
NaCl [34]	mp‐22862	2	259.370	1.560	46.096	58.443	7.962	7.1
NaF [34]	mp‐682	2	319.147	1.500	25.894	41.988	9.580	18.4
NaI [34]	mp‐23268	2	111.927	1.560	69.675	149.894	1.883	1.8
PbS [34]	mp‐21276	2	166.105	2.000	54.174	239.265	5.251	2.9
RbBr [34]	mp‐22867	2	111.057	1.450	86.781	165.372	2.562	3.8
RbCl [34]	mp‐23295	2	130.816	1.450	75.148	120.921	2.919	2.8
RbI [34]	mp‐22903	2	95.800	1.410	104.957	212.372	2.392	2.3
SrO [34]	mp‐2472	2	314.828	1.520	35.277	103.619	24.442	12
PbTe [34]	mp‐19717	2	120.566	1.450	70.758	334.800	6.201	2.5
ZnS [34]	mp‐10695	2	276.738	1.685(By Equation (7))	40.476	97.474	13.047	27
ZnSe [34]	mp‐1190	2	227.402	1.727(By Equation (7))	47.338	144.369	10.705	19
ZnTe [34]	mp‐2176	2	184.622	0.970	59.146	193.009	28.592	18
AlN [34]	mp‐661	4	613.928	0.700	42.527	81.978	374.316	350
BeO [34]	mp‐2542	4	697.821	0.750	27.992	50.022	258.052	370
CdS [34]	mp‐672	4	136.777	0.750	104.863	288.952	17.433	16
GaN [34]	mp‐804	4	395.693	0.700	46.943	167.460	211.580	210
SiC [34]	mp‐7140	4	785.624	0.750	42.006	80.194	675.863	490
ZnO [34]	mp‐2133	4	225.909	0.750	49.719	162.816	34.512	60

Having established the robustness of the validation tools, we benchmarked the efficacy of the LLM‐assisted re‐ranking strategy against a BO‐only baseline without evidence‐conditioned re‐ranking. As shown in Figure 3a, the LLM‐assisted strategy yields a higher cumulative number of validated low‐κ candidates than the BO‐only baseline over 20 iterations. Specifically, the LLM‐assisted re‐ranking yields 221 cumulative validated low‐κ candidates, compared with 155 for the BO‐only baseline. The advantage becomes more pronounced in later iterations. Notably, this discovery trajectory accelerates in the later stages, reflecting the benefit of combining BO acquisition ranking with local, mechanism‐aware re‐ranking before expensive validation. Beyond quantity, Figure 3b compares the κ distributions of the validated candidates and shows that the LLM‐guided workflow maintains a strong yield across the low‐κ search window while increasing the total number of successful candidates. This comparison shows that qualitative LLM re‐ranking improves the yield of candidates that pass the downstream validation pipeline. Stability and low‐κ success are determined only after structure generation, relaxation, phonon calculations, and κ evaluation. All dynamically stable low‐κ materials identified by the LLM‐BO and BO‐only methods are available in the supplementary file “lowKappa_Materials.zip”. We further examined the crystallographic symmetry distribution of the generated candidates to determine whether the previously observed dominance of P1 structures originated from intrinsic material characteristics or from unconstrained structure generation. The analysis indicated that the strong P1 preference in the original candidates primarily arose from the lack of explicit symmetry guidance during crystal structure generation.This behavior is consistent with previous reports that symmetry‐agnostic crystal‐generation models tend to overproduce P1 or other low‐symmetry structures [37, 38]. To mitigate this crystallographic bias, we introduced symmetry‐aware structural diversification during LLM‐guided candidate expansion. The revised framework achieves a broader symmetry distribution while preserving low‐symmetry candidates that may be relevant for ultralow‐ materials exploration (Figure S17). The new dynamically stable low‐κ materials identified by the symmetry‐aware structural diversification methods are available in the supplementary file “lowKappa_symmetry_aware_Materials.zip”.

**FIGURE 3:**
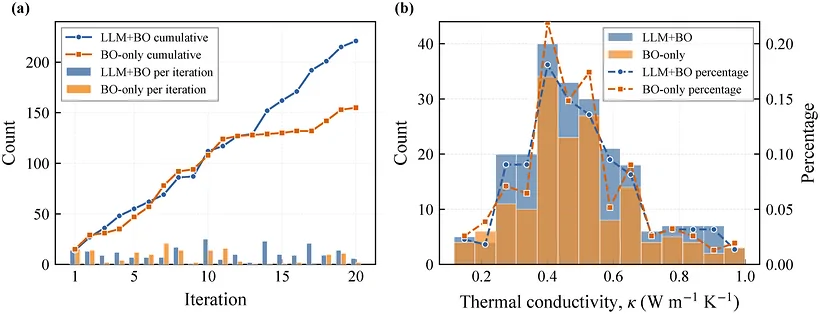
Benchmarking the efficiency of the LLM‐assisted re‐ranking strategy. (a) Discovery trajectory over 20 iterations. Comparison of cumulative (solid lines) and per‐iteration (bars) discovery counts between the LLM‐guided Bayesian optimization (LLM‐BO) and the conventional baseline (BO‐only). The LLM‐assisted re‐ranking workflow yields a higher cumulative yield in identifying low‐κ candidates. (b) Distribution of discovered materials. Histograms showing the count (bars) and percentage distribution (dashed lines) of discovered candidates as a function of predicted κ at 300 K. The LLM‐guided strategy increases the number of validated low‐κ candidates while preserving a broad distribution across the target range below 1.0 W $m^{-1}$ $K^{-1}$, supporting its role as a prioritization layer before deterministic physics‐based validation.

To substantiate the predictive fidelity of our framework and elucidate the microscopic origins of ultralow κ, we prioritized three standout candidates isolated by the LLM‐assisted prioritization and physics‐validation workflow: Ag_3_SbSe_4_, Ag_4_TiSe_4_, and Ag_4_TiTe_4_. We first examine the structural properties of Ag_3_SbSe_4_, which crystallizes in the cubic *P*‐43*m* space group (No.215). Its primitive cell comprises eight atoms with a lattice constant of a=b=c=6.4449 Å (Figure 4a). Crucially, the complete absence of imaginary frequencies within the harmonic approximation (HA) confirms that Ag_3_SbSe_4_ is dynamically stable at 0 K (Figure 4b). The absence of imaginary phonon modes establishes the dynamical stability of the relaxed structures for the three highlighted candidates. To assess their thermodynamic stability, the energy above the convex hull ($E_{\text{hull}}$) was evaluated using ternary phase diagrams constructed from Materials Project reference phases and our calculated DFT total energies (Figure S4). While ${\text{Ag}}_{4}{\text{TiSe}}_{4}$ and ${\text{Ag}}_{4}{\text{TiTe}}_{4}$ lie relatively close to the convex hull with $E_{\text{hull}}$ values of 0.049 and 0.052 eV/atom, respectively, ${\text{Ag}}_{3}{\text{SbSe}}_{4}$ exhibits a larger hull distance of 0.131 eV/atom. Although these positive $E_{\text{hull}}$ values indicate thermodynamic instability with respect to competing phases, large‐scale data‐mining studies have demonstrated that a substantial proportion of experimentally observed inorganic crystals are metastable [39]. Furthermore, applying a strict cutoff of $E_{\text{hull}}<0.2\;\text{eV/atom}$ would inappropriately exclude a significant fraction of known synthesizable polymorphs [40]. Given that the $E_{\text{hull}}$ values for all three candidates fall well below this 0.2 eV/atom threshold, they possess thermodynamic accessibility to exist as metastable phases. Therefore, rather than claiming them as confirmed thermodynamically stable phases, we retain them as dynamically stable, low‐κ metastable candidates whose successful realization remains subject to explicit experimental validation.

**FIGURE 4:**
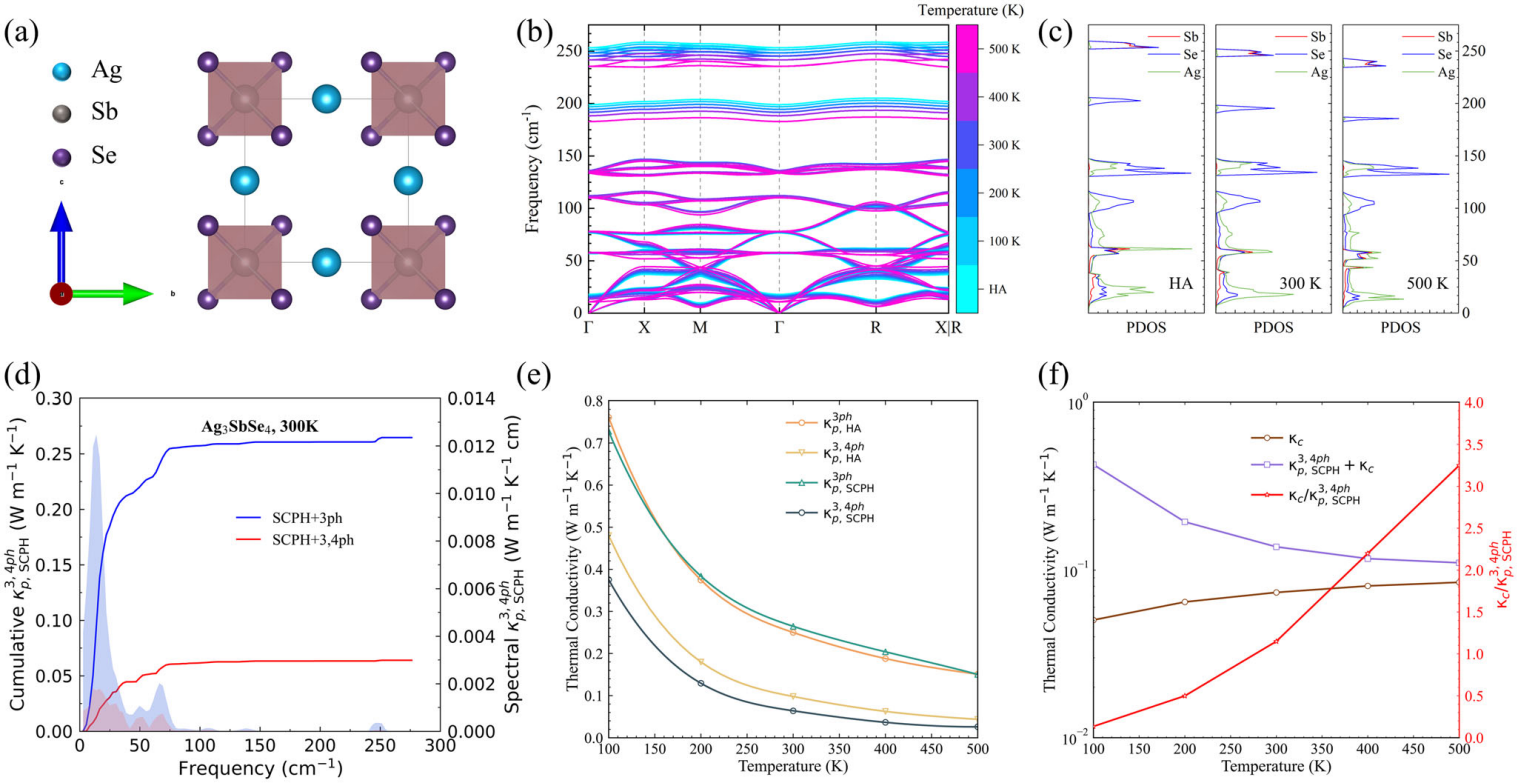
(a) The primitive cell structure of Ag_3_SbSe_4_. (b) The temperature‐dependent phonon spectrum from zero to 500 K for Ag_3_SbSe_4_. (c) Projected phonon density of states (PDOS) of Ag_3_SbSe_4_ at 0 K under harmonic approximation (HA), 300, and 500 K. (d) Calculated spectral and cumulative $\kappa_{p,\;SCPH}^{3,4ph}$ using the SCPH+3,4ph model at 300 K. (e) Temperature dependence of the phonon populations' thermal conductivity ($\kappa_{p}$). (f) Intrinsic κ, coherent phonon thermal conductivity ($\kappa_{c}$) and $\kappa_{c}/\kappa_{p,\;SCPH}^{3,4ph}$ (right axis) as a function of the temperature of Ag_3_SbSe_4_.

To quantify the impact of higher‐order terms on thermal transport, we examined the temperature‐dependent phonon spectra incorporating fourth‐order anharmonicity. Using interatomic force constants (IFCs) derived from the advanced compressive sensing lattice dynamics (CSLD) method [41, 42], we mapped the phonon spectral evolution as a function of temperature (Figure 4b,c). Upon heating from 0 to 500 K, the phonon spectrum of Ag_3_SbSe_4_ exhibits a complex renormalization behavior, characterized by the coexistence of hardening and softening in low‐frequency acoustic phonon modes, contrasted with the consistent softening of high‐frequency optical branches. This pronounced spectral shift serves as a hallmark of strong lattice anharmonicity in Ag_3_SbSe_4_, demonstrating that accounting for temperature‐induced phonon renormalization is indispensable for accurate thermal predictions.

Dissecting the spectral contributions reveals that low‐frequency phonons below 60 ${\mathrm{cm}}^{-1}$ are heavily dominated by the vibrations of heavy Ag atoms, as evidenced by the projected phonon density of states (PDOS) in Figure 4c. Phonon transport calculations based on Peierls–Boltzmann theory (Figure 4d) further show that thermal transport at 300 K is overwhelmingly sustained by these low‐energy phonon modes. Quantitatively, phonons with frequencies below 75 ${\mathrm{cm}}^{-1}$ are the primary heat carriers. Generally, κ is the summation of the Peierls contribution from diagonal term $\kappa_{p}$ and the Wigner distribution from off‐diagonal term $\kappa_{c}$, $\kappa_{L}=\kappa_{p}+\kappa_{c}$, wherein the $\kappa_{c}$ originates from the Wigner distribution associated with the wave‐like tunneling [43, 44, 45]. Notably, phonons below 40 ${\mathrm{cm}}^{-1}$ account for 69% of the total $\kappa_{p}$ under the SCPH+3ph approximation. When four‐phonon scattering is included (SCPH+3,4ph), this relative contribution rises to 80%. This shift indicates that higher‐order anharmonicity disproportionately suppresses high‐frequency optical channels, leaving low‐frequency acoustic modes driven by Ag dynamics as the dominant drivers of thermal transport in Ag_3_SbSe_4_.

Figure 4e compares κ derived from three theoretical hierarchies. Given the cubic symmetry of Ag_3_SbSe_4_, thermal transport is isotropic. Initially, both the harmonic approximation (HA+3ph) and the more rigorous self‐consistent phonon model (SCPH+3ph) yield comparable magnitudes: 0.7‐0.8, 0.25, and 0.15 W $m^{-1}$ $K^{-1}$ at 100, 300, and 500 K, respectively. This similarity arises from the fortuitous cancellation of phonon hardening and softening effects [46], consistent with the competing frequency shifts observed in Figure 4b. Including four‐phonon scattering (SCPH+3,4ph) precipitates a collapse in $\kappa_{p}$ to 0.38, 0.064, and 0.026 W $m^{-1}$ $K^{-1}$ at 100, 300, and 500 K. These values represent a staggering 48%–83% suppression relative to the SCPH+3ph baseline and a 22%–41% reduction compared to HA+4ph results. These distinct deviations underscore a critical finding: accurate thermal transport prediction in such highly anharmonic systems necessitates the simultaneous treatment of both temperature‐dependent phonon renormalization and higher‐order four‐phonon scattering.

Beyond the particle‐like scattering picture, we find that wavelike coherence contributions are non‐negligible and substantial in the thermal transport of complex materials. Incorporating the coherence term ($\kappa_{c}$) alongside the particle term ($\kappa_{p,SCPH}^{3,4ph}$) significantly elevates the total κ as shown in Figure 4f. Remarkably, the ratio $\kappa_{c}/\kappa_{p,SCPH}^{3,4ph}$ surpasses 1.0 at 300 K and escalates with temperature, reaching 3.25 at 500 K. Consequently, the off‐diagonal coherence channel effectively supplants particle diffusion as the dominant transport mechanism above room temperature. Although the thermal transport in Ag_3_SbSe_4_ phenomenologically mimics that of amorphous materials, this “glass‐like” behavior arises fundamentally from strong phonon anharmonicity, distinct from the structural disorder mechanism observed in traditional glasses [43, 44, 45]. To complete the thermodynamic characterization and contextualize the ultralow κ, we analyzed the specific heat capacity ($C_{V}$). $C_{V}$ asymptotically approaches the theoretical Dulong–Petit limit of 0.262 J $g^{-1}$ $K^{-1}$ above 300 K. This value sits between that of liquid‐like Cu_2_Se (0.36 J $g^{-1}$ $K^{-1}$) and PbTe (0.156 J $g^{-1}$ $K^{-1}$), confirming typical thermodynamic behavior and isolating strong anharmonic scattering as the primary driver of the ultralow conductivity of Ag_3_SbSe_4_.

Further analysis of key transport descriptors (Figure 5a–d) reveals that the ultralow κ stems primarily from intrinsically suppressed phonon group velocities. Given that κ scales with the square of the group velocity ($\upsilon^{2}$), we mapped its frequency dependence at 300 K (Figure 5b). The results are striking: the maximum $\upsilon^{2}$ for Ag_3_SbSe_4_ is merely 0.97 ${\mathrm{km}}^{2}$ $s^{-2}$. This value stands in stark contrast to the ∼ 14 ${\mathrm{km}}^{2}$ $s^{-2}$ maximum observed in the benchmark thermoelectric PbTe [47]. Such a drastic reduction identifies these suppressed phonon velocities as the decisive factor driving the ultralow κ.

**FIGURE 5:**
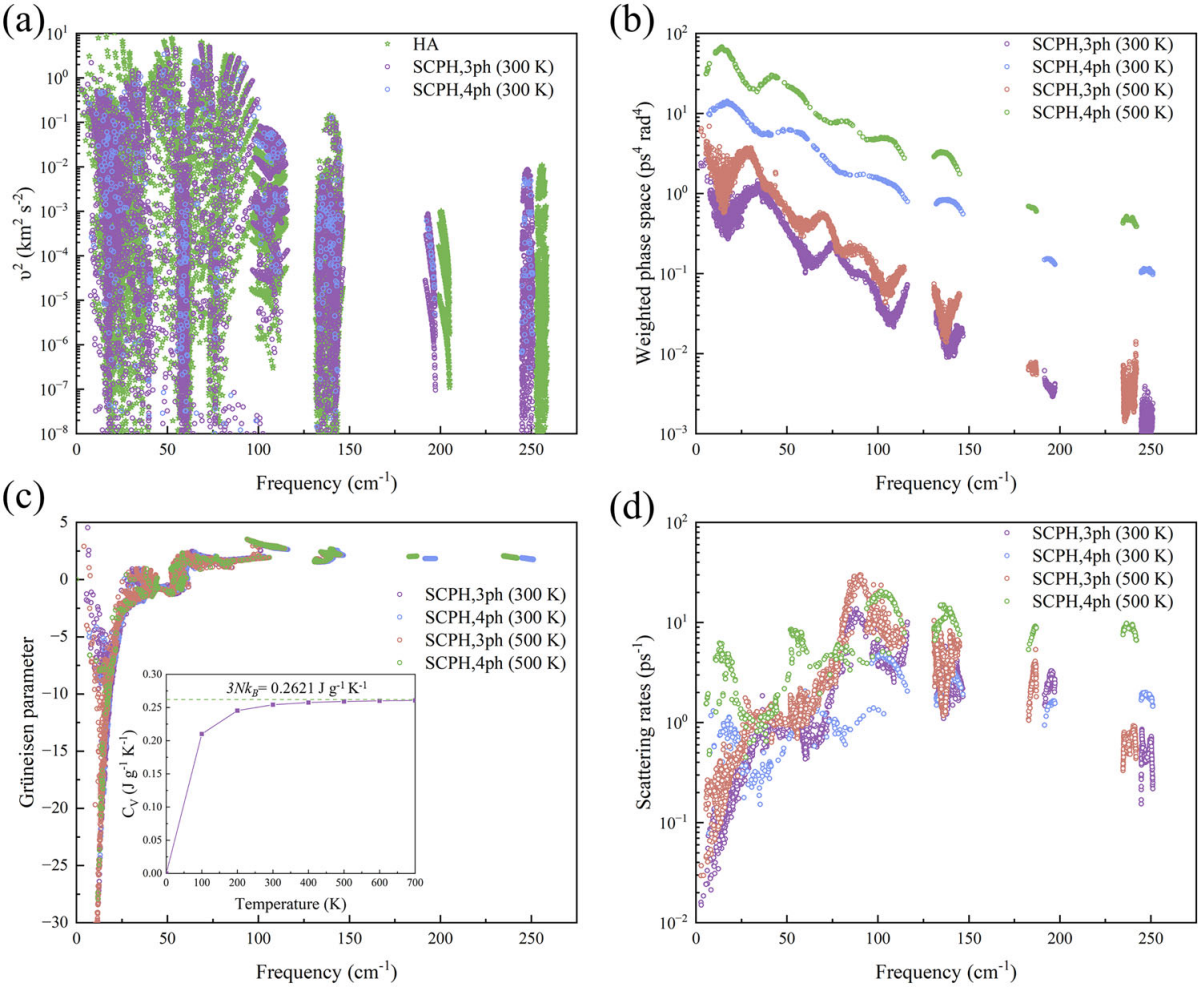
(a) Squared phonon velocities of Ag_3_SbSe_4_ in the harmonic approximation and the anharmonic renormalization approximation at 300 K. (b) Weighted phonon scattering phase space of 3ph and 4ph for Ag_3_SbSe_4_ at 300 and 500 K. The inset is specific heat capacity at constant volume ($C_{V}$). (c) The Grüneisen parameter of 3ph and 4ph for Ag_3_SbSe_4_ at 300 and 500 K. (d) Phonon scattering rates of 3ph and 4ph for Ag_3_SbSe_4_ at 300 and 500 K.

The physical origin of the prevailing four‐phonon (4ph) interactions is elucidated by the scattering phase space analysis (Figure 5d). It is evident that the available phase space for 4ph processes ($P_{4}$) is orders of magnitude larger than that for 3ph processes ($P_{3}$) and exhibits a stronger positive correlation with temperature. Crucially, a pronounced peak in $P_{4}$ near 20 ${\mathrm{cm}}^{-1}$ is intrinsically linked to the low‐frequency flat bands. These localized modes drastically expand the available scattering channels, thereby further suppressing κ. This abundance of phase space manifests directly in the remarkably high phonon scattering rates of Ag_3_SbSe_4_. A distinct peak observed around 60 ${\mathrm{cm}}^{-1}$ in the scattering rates aligns perfectly with the flat optical branches (Figure 4b). This correspondence provides concrete evidence that these flat modes act as critical scattering centers, effectively throttling thermal transport by amplifying anharmonic phonon‐phonon interactions.

To probe the chemical bonding nature underlying the anomalous lattice dynamics, we analyzed the Electron Localization Function (ELF) (Figure 6a,b). The maps reveal significant electron delocalization surrounding the Ag atoms, in sharp contrast to the localized states observed around Sb and Se. This distinct delocalization points to a chemically softened bonding environment indicative of strong electron‐phonon coupling [48, 49]. This interpretation is strictly reinforced by the Projected Density of States (PDOS) (Figure 4c), where Ag atoms dominantly populate the soft, low‐frequency phonon modes (<15 and 60 ${\mathrm{cm}}^{-1}$) [50, 51], linking the electronic environment directly to the suppression of thermal transport.

**FIGURE 6:**
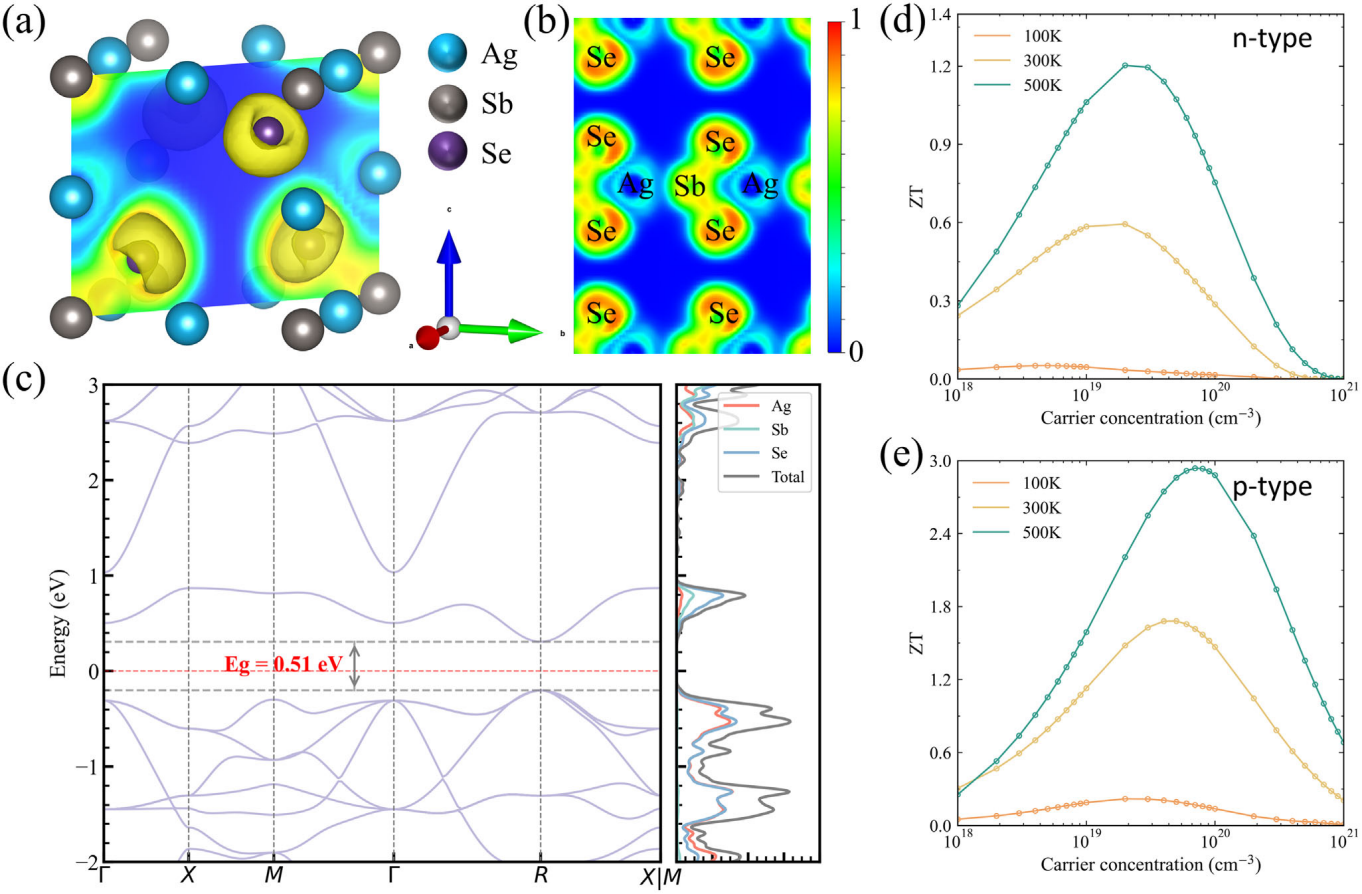
(a) Electron Localization Function (ELF) of Ag_3_SbSe_4_ in (a) 3D and (b) 2D plots. ELF = 1 corresponds to complete localization, and ELF = 0 corresponds to complete delocalization of electrons. (c) Electronic band structure and electronic density of states (DOS) of Ag_3_SbSe_4_. Calculated thermoelectric figure of merit (ZT) of (d) n‐type and (e) p‐type Ag_3_SbSe_4_ as a function of carrier concentrations at different temperatures ranging from 100 to 500 K.

Turning to the electronic transport potential, Figure 6c details the electronic structure of Ag_3_SbSe_4_, identifying it as a direct bandgap semiconductor with a PBE‐calculated gap of 0.51 eV at the R point. Orbital analysis indicates that the conduction band minimum (CBM) is primarily derived from hybridized Sb and Se states, while the Valence Band Maximum (VBM) originates from Ag and Se interactions. Crucially, the electronic architecture exhibits hallmarks of a high‐performance thermoelectric: multiple valley degeneracy at the VBM (specifically at the Γ and R points) facilitates a high density‐of‐states effective mass, while the steep dispersion near the CBM ensures high carrier mobility for electrical conductivity (σ). These synergistic features are favorable for increasing the power factor and, consequently, the figure of merit (ZT), perfectly aligning with established band engineering principles [52, 53].

To translate the favorable electronic architecture into quantitative transport metrics, we calculated the Onsager coefficients incorporating a comprehensive scattering model. Our transport simulations go beyond the constant relaxation time approximation by rigorously accounting for acoustic deformation potential (ADP) [54], polar optical phonons (POP) [55], and ionized impurity (IMP) scattering [56] (Section 3 and Figure S5). Integrating these electronic properties with the ultralow lattice thermal conductivity derived earlier, we mapped the temperature‐ and doping‐dependent ZT (Figure 6d,e). Because the low‐κ screening objective is independent of electronic transport, this ZT analysis is used only as downstream thermoelectric validation of selected low‐κ candidates. ZT is not used in BO acquisition or LLM ranking. The optimization landscape reveals a clear trend: as temperature rises from 100 to 500 K, the ZT peak dynamically shifts toward higher carrier concentrations (1.0 × 10
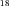
–1.0 × 10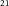 ${\mathrm{cm}}^{-3}$), exhibiting a non‐monotonic dependence on doping levels. Crucially, we identify a robust optimal doping window ranging from 1×10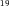 to 7×10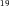 ${\mathrm{cm}}^{-3}$, within which Ag_3_SbSe_4_ achieves its maximal thermoelectric performance, supporting its potential for further thermoelectric evaluation.

A distinct asymmetry exists between the thermoelectric performance of p‐ and n‐type regimes. At 500 K, hole‐doped (p‐type) Ag_3_SbSe_4_ reaches a predicted maximum ZT of 2.94 at a carrier concentration of $7\times 10^{19}$ ${\mathrm{cm}}^{-3}$. This significantly eclipses its n‐type counterpart, which peaks at a modest ZT of 1.2 ($2\times 10^{19}$ ${\mathrm{cm}}^{-3}$), underscoring the critical advantage of leveraging the high valence band degeneracy for hole transport in the 300–500 K range. Furthermore, the downstream transport calculations indicate promising predicted performance in the Ag_4_TiX_4_ (X═Se, Te) family. Our calculations predict peak ZT values at 700 K for the p‐type variants: 2.47 for Ag_4_TiSe_4_ ($2\times 10^{20}$ ${\mathrm{cm}}^{-3}$) and 3.68 for Ag_4_TiTe_4_ ($9\times 10^{19}$ ${\mathrm{cm}}^{-3}$). These predicted ZT values are consistent with the ultralow κ established earlier and the calculated power factors, and they are reported as downstream thermoelectric validation rather than as an objective of BO acquisition or LLM ranking.

## 3 Conclusion

In summary, we developed an LLM‐assisted low‐κ candidate‐prioritization workflow that combines BO‐based composition search, evidence‐conditioned LLM re‐ranking, and deterministic high‐throughput validation. In this workflow, BO acts as the global exploration engine, the LLM re‐ranks only the top 20 BO‐ranked candidates, and final acceptance is determined by structure relaxation, phonon analysis, and κ evaluation. The reliability of our candidates is secured by a comprehensive computational validation suite, uniting CrystaLLM for generative design, MatterSim for dynamical stability verification, and the KappaP rapid κ‐evaluation component for instantaneous property assessment. This workflow culminated in the discovery of three dynamically stable low‐κ Ag‐based chalcogenide candidates, including Ag_3_SbSe_4_, Ag_4_TiSe_4_, and Ag_4_TiTe_4_; downstream electronic‐transport calculations further indicate promising predicted ZT values. The high‐fidelity data generated from deterministic validation are fed back to refine the surrogate model and the structured evidence records used in subsequent re‐ranking cycles.

Broadly, this work underscores the utility of incorporating workflow‐level physical guidance into LLM‐assisted materials‐search pipelines. Unlike traditional “black‐box” approaches that rely solely on statistical correlations, our integration of an LLM‐assisted evidence‐conditioned re‐ranking module incorporates explicit domain evidence, ranging from bonding rules to synthesis feasibility, into candidate prioritization before deterministic validation. This ensures that computational exploration is not only efficient but also physically grounded, significantly mitigating the false‐positive rates endemic to high‐throughput screening. Looking ahead, this paradigm of “LLM‐assisted, physics‐guided search” offers a practical pathway for next‐generation materials design. Future work can extend this workflow by adding validated evidence sources and physics modules for defect chemistry, phase transitions, and synthesis constraints while preserving the separation between BO search, LLM‐assisted prioritization, and physics‐based final validation.

## 4 Experimental Section

### 4.1 Data Preparation

For the elastic modulus data, we collect this data from MatBench [57], which is a machine learning benchmark suite for materials science that includes two elasticity‐related subsets: “matbench_log_gvrh” and “matbench_log_kvrh”. Both subsets contain 10 987 identical materials and are specifically designed for predicting the shear modulus (G) and bulk modulus (B) of materials, respectively, using the Voigt–Reuss–Hill (VRH) averaging scheme and presented on a logarithmic scale. This work utilizes these standardized datasets as our machine learning models training datasets for these critical elastic properties. Figure S2 presents statistics on the number of atoms in the primitive cell, crystal systems, and element distribution in our training dataset. As illustrated in Figure S2, the dataset covers a wide range of atomic numbers, including crystal structures from 7 crystal systems and 84 distinct elements. Additionally, the κ prediction datasets were obtained from the AFLOW database and relevant literature [34, 35], which include numerous experimental values. The AFLOW database offers extensive information on crystal structures and thermal properties. To establish a testing dataset for evaluating the predictive performance of our app, we collected crystal structures, Grüneisen parameters, and corresponding κ values from AFLOW, calculated using the methods described in [10, 58, 59, 60]. In this study, AFLOW is used as an external benchmark for κ prediction. MP, OQMD, and AFLOW entries are not used as the source of the current BO‐generated candidate pool; they are used for benchmarking, final novelty comparison, and provenance checks after candidate generation and validation.

The initial training dataset for thermal conductivity was curated from the literature [61] and further subjected to a series of preprocessing steps before model development. First, all records were standardized with respect to chemical formula representation, in which the elemental compositions were normalized to a consistent ordering over the selected element set, and the reported temperatures were unified in Kelvin. A preliminary composition‐based screening was then performed to retain only compounds composed exclusively of 14 elements commonly associated with intrinsically low lattice thermal conductivity (κ), namely Ag, As, Bi, Cu, Ge, In, Pb, S, Sb, Se, Sn, Te, Ti, and V. This rigorous selection process initially identified 1,466 candidates. To ensure compatibility with the subsequent KappaP framework, which is optimized for near‐room‐temperature applications, an additional temperature constraint was applied to retain only data points within the range of 295–350 K. For compounds with multiple reported records, only the entries within this temperature interval were retained, yielding 220 compounds that collectively define the compositional search space.After these preprocessing and filtering steps, 220 compounds were retained as the final dataset. Each compound was subsequently represented as a 14‐dimensional compositional vector, in which each dimension corresponds to the atomic fraction of one of the selected elements, thereby defining the machine‐learning input space used for surrogate‐model training and Bayesian optimization.

### 4.2 Feature Representation and Feature‐Set Evaluation

As a check on this design choice, we also carried out an offline feature‐engineering analysis on the 220 samples in the initial dataset. The baseline input was the 14‐dimensional composition vector used above, with entries corresponding to the atomic fractions of Ag, As, Bi, Cu, Ge, In, Pb, S, Sb, Se, Sn, Te, Ti, and V.

From each chemical formula, we generated 46 elemental descriptors from tabulated elemental properties, including atomic mass, atomic radius, electronegativity, valence, group number, and period number. For each property, fraction‐weighted statistics were calculated, including the mean, variance, standard deviation, minimum, maximum, and range. We also included several global composition descriptors, such as the number of elements, total atom count, maximum and minimum atomic fractions, atomic‐fraction standard deviation, configurational entropy, and simple mixing‐related terms.

Before model evaluation, invalid, constant, and duplicated descriptors were removed. Pairwise descriptor correlations were then used to reduce redundancy: when two descriptors had an absolute correlation coefficient greater than 0.85, we retained the one with the stronger correlation to the target thermal conductivity. This step reduced the elemental descriptor pool from 46 to 23 descriptors. The remaining descriptors were ranked using SHAP values from an Extra Trees regression model. Ranked descriptors were then added incrementally and evaluated by ten‐fold cross‐validation; the subset with the lowest cross‐validation RMSE was selected. This procedure yielded four selected elemental descriptors: Radius_max, Group_variance, Radius_range, and Eneg_variance.

Finally, we compared three input representations under the same cross‐validation protocol: the original 14 composition features, the four selected elemental descriptors, and the combined composition‐plus‐elemental‐descriptor representation. As shown in Figure S3, the composition‐only representation gives the best cross‐validation performance among the three tested feature sets. For the composition‐only representation, ten‐fold cross‐validation gives $R^{2}=0.631$, MAE = 0.778 W $m^{-1}$ $K^{-1}$, and RMSE = 2.600 W $m^{-1}$ $K^{-1}$, supporting its use as the BO input. We therefore use composition features to train the model in the pre‐generation BO stage.

### 4.3 Bayesian Optimization

We employ Bayesian optimization (BO) as the core search strategy to efficiently explore the compositional space for low‐κ materials. BO is a sequential optimization framework that has proven effective for optimizing expensive black‐box functions in materials science [62]. In the present workflow, BO is the global exploration engine that generates and quantitatively ranks candidate compositions before any LLM‐assisted re‐ranking is applied. In our implementation, each candidate composition is encoded as a 14‐dimensional element‐count vector (fixed element library), and the search is constrained to integer stoichiometries with a maximum of 20 atoms per formula unit. The general sampler supports ternary formulas; in the chalcogenide search reported here, candidates are drawn mainly from an A–B–Ch constrained composition subspace, where the A‐site elements are selected from {Ag, Cu, In, Sn, Pb}, the B‐site elements from {As, Sb, Ge, Bi, Ti, V}, and the chalcogen site from {S, Se, Te}. The stoichiometric coefficients are further restricted to 2≤A≤10, 1≤B≤10, and 1≤Ch≤10, subject to the overall constraint that the total number of atoms does not exceed 20 per formula unit. The BO framework uses a Gaussian Process Regression (GPR) surrogate [31] to model y=logκ rather than raw κ, improving numerical stability for strictly positive and skewed targets. Input features are standardized using a StandardScaler. The GPR kernel is defined as

$$K=C\cdot K_{\mathrm{RBF}}+K_{\mathrm{RQ}}+K_{\mathrm{White}},$$

where $K_{\mathrm{RBF}}$ captures smooth trends, $K_{\mathrm{RQ}}$ captures multi‐scale variation, and $K_{\mathrm{White}}$ models observation noise. We split data into 90% train/validation and 10% test sets; 10‐fold cross‐validation is performed on the train/validation split, and the best‐fold model is retained for BO scoring. Candidate selection is guided by the Expected Improvement (EI) acquisition function, expressed as

$$EI\left(x\right)=\left(f_{\min}-\mu \left(x\right)-\xi \right)\Phi \left(Z\right)+\sigma \left(x\right)\phi \left(Z\right),$$

$$Z=\frac{f_{\min}-\mu \left(x\right)-\xi }{\sigma (x)},$$

where $f_{\min}$ denotes the lowest observed κ, while μ(x) and σ(x) represent the GPR‐predicted mean and standard deviation at composition x, respectively. The exploitation term $(f_{\min}-\mu \left(x\right)-\xi )\cdot \Phi \left(Z\right)$ prioritizes regions with low predicted values, whereas the exploration term σ(x)·ϕ(Z) encourages sampling in uncertain regions. Maximizing EI balances these competing objectives, preventing premature convergence to local minima.

To accommodate the discrete combinatorial nature of chemical formulas, candidate generation is performed via a Metropolis–Hastings MCMC sampler operating in the constrained ternary composition space (maximum atom count enforced). Proposal moves include element substitution, stoichiometric ±1 perturbations, and ternary switches. Each proposal is scored by a weighted objective that integrates EI, predictive uncertainty, and the GPR‐estimated κ, and acceptance follows the standard Metropolis–Hastings criterion. When historical successful or dynamically stable cases are available, the chain is initialized from a randomly selected seed composition; otherwise it starts from a randomly generated ternary composition.

The framework operates in active learning mode wherein validated candidates are iteratively incorporated into the training set to refine the GPR surrogate model. This closed‐loop mechanism progressively sharpens the model's response surface representation, enabling adaptive evolution from broad exploration to targeted optimization.

### 4.4 AI‐guided Material Evaluation and Domain‐Knowledge Update

To enhance the efficiency of material discovery, we integrate an LLM‐assisted evidence‐conditioned re‐ranking module to evaluate candidates recommended by Bayesian optimization (as illustrated in Figure 1). The 20 highest‐scoring compositions ranked by BO/EI are submitted to this re‐ranking module. The LLM is restricted to post‐BO re‐ranking and does not alter the candidate generation process. It operates exclusively on the top 20 BO‐ranked candidates and does not participate in global search. In this implementation, we use DeepSeek; the same workflow can be applied with other LLM backends, such as GPT, Gemini, or Grok, provided that the same evidence‐recording and deterministic validation protocol is retained. The module operates through automated analysis of theoretical principle documents that encode domain knowledge on factors affecting lattice thermal conductivity, including lone pair electron effects [63], mass‐contrast scattering [34], lattice anharmonicity, phonon–phonon interactions, and crystal structure complexity.

Beyond the internal assessment knowledge base, the re‐ranking module is integrated with a controlled public web‐search evidence‐enrichment step implemented through Agno WebSearchTools [33] to perform comprehensive feasibility assessments. The web‐search step relies on publicly accessible search results to provide supplementary evidence, without accessing restricted or subscription‐based content. For each candidate, the module assembles evidence on: (1) chemical composition compatibility with low‐κ design principles, (2) electronic structure features (e.g., presence of heavy elements, electronegativity differences), (3) structural characteristics that promote phonon scattering (e.g., complex unit cells, atomic mass disorder), and (4) historical precedents from reported thermoelectric materials. This evidence fusion supports an evidence‐conditioned re‐ranking of the top 20 BO‐ranked compositions and retains the top 10 compositions for structure generation, accompanied by detailed theoretical justifications for each selection. This local re‐ranking step significantly reduces computational costs by filtering out theoretically unfavorable compositions before expensive structure generation and phonon calculations. For auditability, the workflow stores generated queries, extracted source links, summaries/snippets, retrieval errors, candidate scores, selected materials, and the selection_trace as structured artifacts. If retrieval fails, the error is recorded and the workflow falls back to the fixed screening logic rather than silently changing the ranking protocol.

The framework employs an active learning mechanism where computational results are continuously fed back to refine the GPR surrogate and the structured evidence records. Materials meeting the success criteria—dynamically stable structures (no imaginary phonon frequencies) with $\kappa_{L}<1.0$ W $m^{-1}$ $K^{-1}$—are designated as “successful cases” and used to update both the theoretical principle documents and the GPR training dataset. To ensure continuous iteration even when no materials meet the strict criteria, we adopt a “near‐success” fallback strategy: structures with no imaginary frequencies and $\kappa_{L}<5.0$ W $m^{-1}$ $K^{-1}$ are also incorporated into the feedback loop. This closed‐loop knowledge update mechanism updates the data and evidence available to subsequent BO and LLM‐assisted re‐ranking cycles, while final acceptance remains controlled by the deterministic validation criteria described below.

### 4.5 Structure Generation, Calculation and Screening

The top 10 compositions selected by the LLM‐assisted re‐ranking module are processed through an automated computational pipeline to evaluate their dynamical stability and thermal properties. Crystal structure generation is performed using CrystaLLM [23], a Transformer‐based generative model that produces chemically valid crystal structures from composition inputs. In the default pipeline, CrystaLLM generates five candidate crystal structures for each selected composition. CrystaLLM converts the chemical formula of each candidate into a crystallographic information file (CIF) by predicting sequential tokens based on learned patterns from thousands of known inorganic structures. The generated CIF files are normalized using the built‐in reconstruction module to ensure standard formatting. Duplicate candidates and outputs are controlled at multiple stages: formula‐level duplicate generation is avoided during sampling, relaxed generated structures are deduplicated using pymatgen's StructureMatcher, successful/dynamically stable outputs are deduplicated before feedback, and final validated structures are compared with MP/OQMD/AFLOW records to assess structural novelty.

Structural optimization is carried out using MatterSim [20], a universal machine learning interatomic potential trained across diverse elements, temperatures, and pressures. The relaxation process employs a two‐stage strategy to ensure convergence to relaxed local‐minimum geometries. In the coarse relaxation stage, we apply the Broyden–Fletcher–Goldfarb–Shanno (BFGS) algorithm with the exponential cell filter (ExpCellFilter), enabling simultaneous optimization of atomic positions and lattice parameters. Convergence criteria are set to a maximum force of 0.05 eV/Å, a maximum atomic displacement of 0.1 Å per step, and a maximum of 500 iterations. The second stage performs fine relaxation with more stringent thresholds: maximum force of 0.01 eV/Å, displacement step of 0.05 Å, and up to 500 iterations. This hierarchical approach balances computational efficiency with structural accuracy.

Phonon spectrum calculations are performed using the finite displacement method implemented in MatterSim. A 2×2×2 supercell is employed with atomic displacements of 0.01 Å to construct the force constant matrix. Phonon dispersion relations are obtained by solving the lattice dynamics equations. Materials exhibiting imaginary phonon frequencies are rejected as dynamically unstable. For dynamically stable structures, κ is extracted from the phonon properties. We use this criterion to establish dynamical stability; thermodynamic stability would require formation‐energy or convex‐hull evidence and is therefore not inferred from phonon spectra alone. For Ag_3_SbSe_4_, Ag_4_TiSe_4_, and Ag_4_TiTe_4_, DFT total energies were calculated for the relaxed candidate structures using the settings summarized in the Supporting Information. Reference competing phases in the corresponding ternary chemical systems were obtained from the Materials Project. Formation energies and energies above the convex hull were then evaluated from ternary phase diagrams constructed using the combined set of reference phases and candidate entries. These results provide thermodynamic context, whereas dynamical stability is assessed independently from phonon spectra.

The screening process applies hierarchical criteria to identify successful candidates. First‐tier success cases must satisfy: (1) no imaginary frequencies across the entire Brillouin zone, and (2) room‐temperature $\kappa_{L}<1.0$ W $m^{-1}$ $K^{-1}$. When no materials meet these strict thresholds, the criteria are relaxed to define “near‐success” cases: dynamically stable structures with $\kappa_{L}<5.0$ W $m^{-1}$ $K^{-1}$. These thresholds are applied deterministically after structure generation, relaxation, and phonon calculations. The LLM does not apply the final success criteria; these criteria are evaluated only after structure generation, relaxation, phonon calculations, and κ prediction. Both categories of validated materials are fed back into the Bayesian optimization training set and used to update the structured evidence records used by the LLM‐assisted re‐ranking module, enabling iterative refinement of the discovery framework.

### 4.6 KappaP Model for Lattice Thermal Conductivity

For rapid estimation of κ, we employ the Slack model [64], which captures the dominant contribution from acoustic phonon modes in nonmetallic crystals. The empirical expression is given by,

(1) $$\kappa_{L}=A\frac{M\theta_{a}^{3}V^{\frac{1}{3}}}{\gamma^{2}Tn},$$

where M is the total atomic mass, V the primitive cell volume, n the number of atoms per cell, $\theta_{a}$ the acoustic Debye temperature, γ the Grüneisen parameter, and T the absolute temperature [59]. The coefficient A is expressed as [34],

(2) $$A=\frac{2.43\times 10^{-6}}{1-\frac{0.514}{\gamma }+\frac{0.228}{\gamma^{2}}},$$

The key parameters–average sound velocity ($\upsilon_{s}$), acoustic Debye temperature ($\theta_{a}$), Poisson ratio (ν), and Grüneisen parameter (γ)–are derived from the bulk modulus (B) and shear modulus (G) via established relations [65, 66, 67, 68, 69],

(3) $$\begin{array}{cc} \upsilon_{s} & ={\left\{\frac{1}{3}\left[\frac{1}{\upsilon_{l}^{3}}+\frac{2}{\upsilon_{t}^{3}}\right]\right\}}^{-\frac{1}{3}}, \end{array}$$

(4) $$\begin{array}{cc} \upsilon_{l} & =\sqrt{\frac{B+\frac{4}{3}G}{\rho }}, \end{array}$$

(5) $$\begin{array}{cc} \upsilon_{t} & =\sqrt{\frac{G}{\rho }}, \end{array}$$

(6) $$\theta_{a}=\frac{h}{k_{B}}{\left(\frac{3}{4\pi }\cdot \frac{1}{V}\right)}^{\frac{1}{3}}\cdot \upsilon_{s},$$

(7) $$\nu =\frac{x^{2}-2}{2x^{2}-2},\;\;\;\gamma =\frac{3}{2}\left(\frac{1+\nu }{2-3\nu }\right).$$

where $\upsilon_{l}$ and $\upsilon_{t}$ denote the longitudinal and transverse sound velocities, respectively, ρ is the mass density, and $x=\upsilon_{l}/\upsilon_{t}$. This framework enables rapid estimation of κ from elastic moduli alone, bypassing computationally expensive phonon calculations. A detailed derivation is provided in the Supporting Information.

### 4.7 First‐Principles Lattice Thermal Conductivity

For rigorous validation of the Slack model predictions, we calculate κ from first principles using the Peierls–Boltzmann transport equation [70]. The phonon contribution to thermal conductivity is expressed as

(8) $$\begin{array}{c} \kappa_{p}=\frac{\hbar^{2}}{k_{B}T^{2}VN_{0}}\sum_{\lambda }n_{\lambda }\left(n_{\lambda }+1\right)\omega_{\lambda }^{2}v\lambda ⊗v\lambda \tau_{\lambda }, \end{array}$$

where ℏ is the reduced Planck constant, $k_{B}$ the Boltzmann constant, T the temperature, V the primitive cell volume, and $N_{0}$ the number of sampled q‐points in the first Brillouin zone. The mode‐resolved quantities $n_{\lambda }$, $\omega_{\lambda }$, $v_{\lambda }$, and $\tau_{\lambda }$ denote the Bose–Einstein occupation, frequency, group velocity, and phonon lifetime for mode λ (wave vector q, branch s), respectively.

Interatomic force constants (IFCs) are computed using a 2×2×2 supercell for Ag_3_SbSe_4_, Ag_4_TiTe_4_, and Ag_4_TiSe_4_. Second‐order IFCs are obtained via the finite displacement method with a 4×4×4 k‐point mesh. Third‐ and fourth‐order IFCs are extracted from compressive sensing lattice dynamics (CSLD) [41, 42], utilizing 40 uncorrelated configurations selected from 2000‐step ab initio molecular dynamics (AIMD) trajectories at 300 K (4 fs time step). Brillouin zone sampling employs q‐meshes of 6×6×6 for four‐phonon scattering and 16×16×16 for three‐phonon processes.

Phonon frequency renormalization from quartic anharmonicity is treated via the self‐consistent phonon (SCPH) approximation [8, 71], which is essential for materials exhibiting strong anharmonicity and soft modes. The SCPH renormalized frequency satisfies

(9) $$\begin{array}{c} \Omega_{\lambda }^{2}=\omega_{\lambda }^{2}+2\Omega_{\lambda }\sum_{\lambda_{1}}I_{\lambda \lambda_{1}}, \end{array}$$

where $\omega_{\lambda }$ is the harmonic frequency and $\Omega_{\lambda }$ the temperature‐renormalized frequency. The self‐energy term $I_{\lambda \lambda_{1}}$ is computed as

(10) $$\begin{array}{c} I_{\lambda \lambda_{1}}=\frac{\hbar }{8N_{0}}\frac{V^{\left(4\right)}\left(\lambda ,-\lambda ,\lambda_{1},-\lambda_{1}\right)}{\Omega_{\lambda }\Omega_{\lambda_{1}}}\left[1+2n_{\lambda }\left(\Omega_{\lambda_{1}}\right)\right], \end{array}$$

where $V^{\left(4\right)}$ denotes fourth‐order IFCs in reciprocal space and $n_{\lambda }$ is the Bose‐Einstein distribution. Equations (9) and (10) are solved iteratively until self‐consistency is achieved. Thermal expansion effects are neglected by fixing the unit cell volume [72].

Beyond the incoherent phonon population term $\kappa_{p}$, we include the coherent contribution $\kappa_{c}$ arising from wave‐like tunneling between near‐degenerate modes [73]

(11) $$\begin{array}{cc} \kappa_{c}= & \frac{\hbar^{2}}{k_{B}T^{2}VN_{0}}\sum_{q}\sum_{s\neq s^{\prime }}\frac{\omega_{q}^{s}+\omega_{q}^{s^{\prime }}}{2}v_{q}^{s,s^{\prime }}v_{q}^{s^{\prime },s}\\ & \times \frac{\omega_{q}^{s}n_{q}^{s}\left(n_{q}^{s}+1\right)+\omega_{q}^{s^{\prime }}n_{q}^{s^{\prime }}\left(n_{q}^{s^{\prime }}+1\right)}{4{\left(\omega_{q}^{s^{\prime }}-\omega_{q}^{s}\right)}^{2}+{\left(\Gamma_{q}^{s}+\Gamma_{q}^{s^{\prime }}\right)}^{2}}\\ & \times \left(\Gamma_{q}^{s}+\Gamma_{q}^{s^{\prime }}\right), \end{array}$$

in which ℏ is the reduced Planck constant, $k_{B}$ the Boltzmann constant, V the unit cell volume, and $N_{0}$ the total number of sampled phonon wave vectors.

## Conflicts of Interest

The authors declare no conflicts of interest.

## Supporting information


**Supporting File 1**: advs78175‐sup‐0001‐VideoS1.mp4.

**Supporting File 2**: advs78175‐sup‐0002‐VideoS2.mp4.

**Supporting File 3**: advs78175‐sup‐0003‐SuppMat.zip.

**Supporting File 4**: advs78175‐sup‐0004‐SuppMat.zip.

**Supporting File 5**: advs78175‐sup‐0005‐SuppMat.docx.

**Supporting File 6**: advs78175‐sup‐0006‐SuppMat.docx.

## Data Availability

All data generated and analyzed in this study, including the curated 220‐compound seed dataset, feature representations, and active‐learning workflow logs, are available at GitHub: https://github.com/Jack‐Liu0227/Auto_Discover_LowThermalConductivity_Materials. Public elastic‐modulus datasets were obtained from the Matbench repository (matbench_log_gvrh and matbench_log_kvrh) [57]. Crystal structures, phonon spectra, and thermal‐conductivity results for identified low‐$\kappa_{L}$ materials are provided in the Supplementary Materials (lowKappa_Materials.zip and lowKappa_symmetry_aware_Materials.zip). The interactive thermal‐conductivity calculation app is available at https://kappap‐ai.streamlit.app/ , and a user tutorial video for the app is provided as an MP4 file in the Supplementary Materials.
