# Supplementary material for: LLM‐Assisted Design of Ultralow‐κ Chalcogenides
Source: Adv Sci (Weinh). 2026 Oct 15:e78175. Online ahead of print. doi: 10.1002/advs.78175 (PMC13672694; doi:10.1002/advs.78175)
Supplement: Supplementary file 1 [file ADVS-9999-e78175-s002.docx]

LLM-assisted design of ultralow-κ chalcogenides

Yujie Liu,^1, †^ Congran Jia,^2, †^ Yuzhou Hao, ^1,^ Xuejie Li, ^1,^ Yang Wang,^1^ Artem Oganov,^3^ Jun Sun,^1^ Xiangdong Ding,^1^ Turab Lookman,^1,4,*^ and Zhibin Gao^1, ‡^

*^1^State Key Laboratory for Mechanical Behavior of Materials, State Key Laboratory of Porous Metal Materials, School of Materials Science and Engineering, Xi’an Jiaotong University, Xi’an, 710049, China*

*^2^School of Physics, Xi’an Jiaotong University, Xi’an, 710049, China*

*^3^Skolkovo Institute of Science and Technology, 30 Bolshoy Boulevard, Bld. 1, Moscow 121205, Russia*

*^4^AiMaterials Research LLC, Santa Fe, New Mexico 87501, United States*

^†^ These authors contributed equally to this work.

^*^E-mail: [turablookman@gmail.com](mailto:turablookman@gmail.com)

^‡^E-mail: [zhibin.gao@xjtu.edu.cn](mailto:zhibin.gao@xjtu.edu.cn)

**Outline**

[1. Method	2](#_Toc233150790)

[1.1 CGCNN method	2](#_Toc233150791)

[1.2 Evaluation metrics	2](#_Toc233150792)

[1.3 Slack model	3](#_Toc233150793)

[1.4 Deployment of KappaP code for thermal conductivity (κ)	5](#_Toc233150794)

[1.5 Lattice thermal conductivity	6](#_Toc233150795)

[1.6 DFT calculation	8](#_Toc233150796)

[1.7 Data collection of elastic modulus	8](#_Toc233150797)

[1.8 Feature-set evaluation for BO surrogate	9](#_Toc233150798)

[1.9 Energy above the convex hull and thermodynamic context	10](#_Toc233150799)

[2. Comparison of calculation time for κ_L_	10](#_Toc233150800)

[3. Electronic transport of Ag_3_SbSe_4_	11](#_Toc233150801)

[4. The first-principles calculation of Ag_4_TiX_4_ (X=Se/Te)	12](#_Toc233150802)

# 1. Method

## 1.1 CGCNN method

CGCNN is an advanced machine learning tool that utilizes trained models to efficiently predict material properties. It transforms crystalline structures into graphical representations, where atoms are depicted as nodes and the bonds between them as edges. This approach allows the network to learn about the local chemical environment of the material.

CGCNN autonomously extracts essential features that enable accurate predictions of various material properties, such as shear and bulk modulus, through the use of convolutional and pooling layers. These predictions are not only highly accurate but also interpretable, providing valuable chemical insights that can guide material design. Additionally, CGCNN exhibits strong generalization capabilities, making it suitable for a diverse range of crystal structures and chemical compositions. This versatility significantly accelerates the discovery and design of new materials^[1–3]^. In this study, the elastic modulus dataset was divided into training, validation, and test sets in an 8:1:1 ratio. The model was trained for 30 epochs using the Adam optimizer with a learning rate of 0.001. The architecture consisted of three convolutional layers and two hidden layers.

## 1.2 Evaluation metrics

In machine learning and statistical analysis, Mean Absolute Error (MAE) and $R^{2}$ (R-squared) are commonly used metrics for evaluating the performance of regression models. These metrics measure prediction accuracy and the model’s explanatory power, respectively.

Mean Absolute Error (MAE) measures the average magnitude of prediction errors, regardless of their direction. It is calculated as:

$$\begin{aligned} \text{MAE}=\frac{1}{n}\sum_{i=1}^{n} \left| y_{i}-\hat{y_{i}} \right|\#\left( 1 \right) \end{aligned}$$

where $y_{i}$ represents the actual values and $\hat{y_{i}}$ represents the predicted values. A lower MAE indicates more accurate predictions. R-squared (R^2^) evaluates the proportion of variance in the dependent variable that is predictable from the independent variables. It is given by:

$$\begin{aligned} R^{2}=1-\frac{\sum_{i=1}^{n} \left( y_{i}-\hat{y_{i}} \right)^{2}}{\sum_{i=1}^{n} \left( y_{i}-\bar{y} \right)^{2}}\#\left( 2 \right) \end{aligned}$$

where $\bar{y}$ is the mean of the actual values. An $R^{2}$ value closer to 1 indicates a better fit of the model to the data.

## 1.3 Slack model

Considering the critical role of acoustic phonon modes in the thermal transport of nonmetallic crystals,$\kappa_{L}$ can be determined using the well-known Slack model^[4]^,

$$\begin{aligned} \kappa_{L}=A\frac{\bar{M}\theta_{a}^{3}\delta n^{1/3}}{\gamma^{2}T}\#\left( 3 \right) \end{aligned}$$

where $\bar{M}$ represents the average atomic mass, $\delta^{3}$ is the volume per atom of the primitive cell, *n* is the number of atoms in the primitive cell, $\theta_{a}$ denotes the acoustic Debye temperature, $\gamma$ is the Grüneisen parameter and *T* is the absolute temperature. Since $\bar{M}=M/n$ and $\delta^{3}=V/n$, the Slack formula can be rewritten in the following,

$$\begin{aligned} \kappa_{L}=A\frac{M\theta_{a}^{3}V^{\frac{1}{3}}}{\gamma^{2}Tn}\#\left( 4 \right) \end{aligned}$$

where M represents the total atomic mass and V is the volume of the primitive cell. Typically, the coefficient A in the Slack model of Eq. (4) is calculated as^[5]^,

$$\begin{aligned} A=\frac{2.43\times{10}^{-6}}{1-\frac{0.514}{\gamma}+\frac{0.228}{\gamma^{2}}}\#\left( 5 \right) \end{aligned}$$

The formula proposed by Slack has been widely used for evaluating $\kappa_{L}$ in numerous materials. A key aspect of $\kappa_{L}$ assessment is determining the acoustic Debye temperature ($\theta_{a}$) and the Grüneisen parameter ($\gamma$).

Jia et al.^[6]^ suggested that the speed of sound can be effectively estimated from the elastic properties--specifically, the bulk modulus (B) and shear modulus (G). This approach is more computationally feasible compared to experimental methods or expensive lattice dynamic simulations. B and G are obtained based on a trained CGCNN model^[1]^. This method offers a computationally alternative for estimating elastic properties, thereby streamlining the value of sound velocities, as demonstrated in^[6,7]^,

$$\begin{aligned} \upsilon_{s}=\left\{ \frac{1}{3}\left[ \frac{1}{\upsilon_{l}^{3}}+\frac{2}{\upsilon_{t}^{3}} \right] \right\}^{-\frac{1}{3}}\#\left( 6 \right) \end{aligned}$$

$$\begin{aligned} \upsilon_{l}=\sqrt{\frac{B+\frac{4}{3}G}{\rho}}\#\left（ 7 \right） \end{aligned}$$

$$\begin{aligned} \upsilon_{t}=\sqrt{\frac{G}{\rho}}\#\left( 8 \right) \end{aligned}$$

where $\upsilon_{s}$, $\upsilon_{l}$, and $\upsilon_{t}$ are the average speed of sound, sound velocities of the longitude and transverse waves in the elastic mechanics, respectively. $\rho$ is the density of the material. With $\upsilon_{s}$, the acoustic Debye temperature ($\theta_{a}$) can be evaluated^[8]^,

$$\begin{aligned} \theta_{a}=\frac{h}{k_{B}}\left( \frac{3}{4\pi}\cdot\frac{1}{V} \right)^{\frac{1}{3}}\cdot\upsilon_{s}\#\left( 9 \right) \end{aligned}$$

After approximating the acoustic Debye temperature using B and G, the next step is to determine the Grüneisen parameter ($\gamma$), which characterizes the anharmonicity of the material^[9]^. Sound velocity serves as an indicator of the strength of harmonic inter-atomic interactions. Weaker interactions generally correspond to lower sound velocities.

Previous studies have shown that the relationship between Poisson’s ratio ($\nu$) and $\gamma$can be expressed as^[10,11]^,

$$\begin{aligned} \nu=\frac{x^{2}-2}{2x^{2}-2},\gamma=\frac{3}{2}\left( \frac{1+\nu}{2-3\nu} \right)\#\left( 10 \right) \end{aligned}$$

where x is the ratio of longitudinal sound velocity to transverse sound velocity, namely $x=\upsilon_{l}/\upsilon_{t}$.

Using the aforementioned approximation method, $\theta_{a}$ and $\gamma$ can be rapidly estimated from elastic properties, specifically the shear modulus and bulk modulus. Previous studies have shown that this method aligns well with experimental values for cubic, isotropic, and quasi-isotropic structures^[6,12,13]^. Note that it may introduce large errors when applied to highly anisotropic systems with low-symmetry lattices, such as triclinic lattices with abnormal atomic arrangements that create large void spaces.

## 1.4 Deployment of KappaP code for thermal conductivity (κ)

FIG. S1 The web page for our prediction app, Kappa Predictor (KappaP), features a layout where the left panel provides the file upload area, while the right panel displays the results. The output begins with a DataFrame that includes the lattice constants for each crystal structure along with additional details. The columns include the number of atoms, density (g cm^−3^), volume (Å^3^), total atomic mass (amu.), bulk modulus (GPa), shear modulus (GPa), sound velocity of the transverse wave (m s^-1^), sound velocity of the longitudinal wave (m s^-1^), speed of sound (m s^-1^), Poisson's ratio (ν), Grüneisen parameter (γ), acoustic Debye temperature (θ_a_) (K), and lattice thermal conductivity (W m^−1^ K^−1^). For guidance on how to use KappaP, please refer to the tutorial video: [KappaP_tutorial.mp4](https://github.com/Jack-Liu0227/AI4Kappa/tree/master/KappaP_Supporting_Information). Additionally, our app supports customized functions for bulk modulus, shear modulus, and Grüneisen parameters. A tutorial for this feature can be found in the video titled [KappaP_Custom_Parameters_tutorial.mp4](https://github.com/Jack-Liu0227/AI4Kappa/tree/master/KappaP_Supporting_Information)

Streamlit is an open-source Python library that simplifies the creation and sharing of custom web applications. It provides a quicker way to build and share data-driven apps, allowing users to transform data scripts into shareable web applications in just minutes. To deploy an app, you first upload the project code to GitHub. Then, using Streamlit, you create a new app by selecting the appropriate project branch and the main Python file (e.g., app.py) to run. This enables you to deploy an app entirely in Python, without needing any front-end experience. Utilizing the Slack model and CGCNN, we developed the κ_L_ calculation app using this framework. If you have the source code, you can deploy the κ_L_ app on your computer or Streamlit’s server. For more details about our software, please refer to the [README.md](https://github.com/Jack-Liu0227/AI4Kappa/blob/master/README.md).

It is important to note that setting up app environments can be complex. Creating a virtual environment for app dependencies is essential, as running a web app requires numerous Python packages with specific versions. Fortunately, our code streamlines this process by allowing you to include a requirements.txt file, ensuring that all Python dependencies are installed correctly. Once the virtual environment is set up, the next step is to build the framework for running the web app. We initially used a revised CGCNN model to predict the bulk modulus (B) and shear modulus (G) from the CIF files uploaded by users. However, obtaining just B and G is insufficient; additional information must also be extracted from the CIF file, such as total atomic mass, volume, number of atoms, and density. This is accomplished by identifying the elements and their quantities within the CIF file in our code.

After determining these fundamental physical properties, we applied physical formulas to calculate the sound velocity (υ_s_), acoustic Debye temperature (θ_a_) and Grüneisen parameter (γ), which are closely related to κ_L_. Finally, by incorporating these parameters into the Slack model, we estimated the approximate κ_L_ for the material.

At this stage, the entire framework of the app is complete. The results are displayed on the website in the form of a DataFrame, with an option to download them in CSV format. After successfully uploading CIF files, the app quickly calculates the material’s elastic properties, *κ_L_*, and other relevant outputs. The app's interface is illustrated in FIG. S1. Notably, users can upload single or multiple CIF files simultaneously, allowing the entire framework to run at once and provide all results together. Our app, KappaP, is easily accessible via the link: <https://kappap-ai.streamlit.app> whether on your phone or local computer.

## 1.5 Lattice thermal conductivity

The lattice thermal conductivity $(\kappa_{p})$, derived from Peierls-Boltzmann transport theory, can be expressed as^[14]^:

$$\begin{aligned} \kappa_{p}=\frac{\hbar^{2}}{k_{B}T^{2}VN_{0}}\sum_{\lambda} n_{\lambda}\left( n_{\lambda}+1 \right)\omega_{\lambda\boldsymbol{v}}^{2}\lambda\otimes\boldsymbol{v}\lambda\boldsymbol{\tau}_{\lambda}\# \end{aligned}\left( 11 \right)$$

where $\hbar$ is the reduced Planck constant, $k_{B}$ the Boltzmann constant, $T$ the absolute temperature,$V$ the primitive unit cell volume, and $N_{0}$ the number of phonon wave vectors sampled in the first Brillouin zone. The terms $n_{\lambda}$, $\omega_{\lambda}$, $\boldsymbol{v}_{\boldsymbol{\lambda}}$, and $\tau_{\lambda}$ correspond to the phonon occupation number, frequency, group velocity, and lifetime for mode $\lambda$, characterized by wave vector $q$and branch index $s$.

For interatomic force constants (IFCs) calculations, a $2 \times2 \times2$ supercell was employed for Ag_3_SbSe_4_, Ag_4_TiTe_4_, and Ag_4_TiSe_4_. Second-order IFCs were generated using the finite displacement method with a supercell and a $4 \times4 \times4$ k-point grid. Additionally, 2000-step ab initio molecular dynamics (AIMD) simulations were performed at 300 K with a 4 ps step size, followed by the selection of 40 random, uncorrelated configurations. Third- and fourth-order IFCs were then obtained via compressive sensing lattice dynamics (CSLD)^[15,16]^. The *q*-mesh was set to $6 \times6 \times6$for four-phonon (4ph) scattering and $16 \times16 \times16$ for three-phonon (3ph) processes.

To account for phonon frequency shifts due to quartic anharmonicity, the self-consistent phonon (SCPH) approximation^[17,18]^ was applied, improving accuracy for materials with strong anharmonicity and soft phonon modes. The SCPH equation is given by:

$\begin{aligned} \Omega_{\lambda}^{2}=\omega_{\lambda}^{2}+2\Omega_{\lambda\sum_{\lambda_{1}} I_{\lambda\lambda_{1}}}\#\left( 12 \right) \end{aligned}$

where $\omega_{\lambda}$ is the harmonic phonon frequency, and Ω_λ_ represents the temperature-renormalized frequency. The term $I_{\lambda\lambda_{1}}$ is computed as:

$\begin{aligned} I_{\lambda\lambda_{1}}=\frac{\hbar}{8N_{0}}\frac{V^{\left( 4 \right)}\left( \lambda,-\lambda,\lambda_{1},-\lambda_{1} \right)}{\Omega_{\lambda}\Omega_{\lambda_{1}}}\left[ 1+2n_{\lambda}\left( \Omega_{\lambda_{1}} \right) \right]\#\left（ 13 \right） \end{aligned}$

Here, $V^{\left( 4 \right)}$denotes fourth-order IFCs in reciprocal space, and $n_{\lambda}$ follows the Bose-Einstein distribution. Equations (12) and (13) both involve $I_{\lambda\lambda_{1}} a$nd $\Omega_{\lambda}$, enabling an iterative solution of the SCPH equations. Note that thermal expansion effects were excluded in these calculations, as the volume was held fixed^[19]^.

For a more comprehensive thermal conductivity prediction, the coherent phonon contribution $\kappa_{c}$, which accounts for phonon tunneling, was incorporated^[20]^

$$\kappa_{c}=\frac{\hbar^{2}}{k_{B}T^{2}VN_{0}}\sum_{q} \sum_{s\neq s^{'}} \frac{\omega_{q}^{s}+\omega_{q}^{s^{'}}}{2}v_{q}^{s,s^{'}}v_{q}^{s^{'},s}$$

$$\begin{aligned} \times\frac{\omega_{q}^{s}n_{q}^{s}\left( n_{q}^{s}+1 \right)+\omega_{q}^{s^{'}}n_{q}^{s^{'}}\left( n_{q}^{s^{'}}+1 \right)}{4\left( \omega_{q}^{s^{'}}-\omega_{q}^{s} \right)^{2}+\left( \Gamma_{q}^{s}+\Gamma_{q}^{s^{'}} \right)^{2}}\#\left（ 14 \right） \end{aligned}$$

$$\times\left( \Gamma_{q}^{s}+\Gamma_{q}^{s^{'}} \right)$$

In which $\hbar$ is the reduced Planck constant, $k_{B}$ the Boltzmann constant, $V$ the unit cell volume, and $N_{0}$the total number of sampled phonon wave vectors.

## 1.6 DFT calculation

DFT calculations were performed using the Vienna Ab-initio Simulation Package (VASP) with the Projector Augmented-Wave (PAW) method and the Perdew-Burke-Ernzerhof (PBE) functional^[21–24]^. A cutoff energy of 500 eV and a $5 \times5 \times3$ Monkhorst-Pack *k*-grids were selected for the calculation. The convergence criterion for the total energy was set to ${10}^{-8}$ eV, and the convergence threshold for the Hellmann-Feynman force on each atom was set to ${10}^{-4}$ eV/Å. A $2 \times2 \times2$supercell and a $4 \times4\times4$ *k*-grids were used to generate the second-order inter-atomic force constants (IFCs) via the finite displacement method. Subsequently, we ran 2000-step ab initio molecular dynamics (AIMD) at 300 K with 4 ps step size and selected 40 random and incoherent configurations. Then we used compressive sensing lattice dynamics (CSLD) to obtain third- and fourth-order IFCs^[16,25]^. Combining all IFCs together, we can calculate the thermal transport properties of Ag_3_SbSe_4_, Ag_4_TiSe_4_ and Ag_4_TiTe_4_.

## 1.7 Data collection of elastic modulus

FIG. S2 Statistical analysis of the elastic modulus dataset. (a) Distribution of atoms in primitive cells showing the range of atomic counts (1-160 atoms) across the dataset. (b) Crystal system distribution which displays the distribution across 7 crystal systems, with cubic being the most common (3847 structures), followed by tetragonal (2055 structures), while triclinic is the least common (199 structures). (c) Elemental distribution that illustrates the frequency of 84 distinct elements. The dataset encompasses transition metals, main group elements, and rare earth elements, with oxygen showing the highest frequency.

## 1.8 Feature-set evaluation for BO surrogate

To test whether additional elemental descriptors improve the surrogate model, we generated 46 descriptors from elemental properties, including atomic mass, atomic radius, electronegativity, valence, group number, and period number, using fraction-weighted statistics and global composition terms. Invalid, constant, duplicate, and highly correlated descriptors were removed, and the remaining descriptors were ranked using SHAP values from an Extra Trees model. The selected elemental-descriptor set contains Radius_max, Group_variance, Radius_range, and Eneg_variance. As shown in FIG. S3, the composition-only representation gives the highest mean cross-validation performance (R^2^ = 0.631, MAE = 0.778 W m^−1^ K^−1^, and RMSE = 2.600 W m^−1^ K^−1^), compared with elemental descriptors (R^2^ = 0.607, MAE = 0.816 W m^−1^ K^−1^, and RMSE = 2.643 W m^−1^ K^−1^) and the composition-plus-elemental-descriptor representation (R^2^ = 0.601, MAE = 0.817 W m^−1^ K^−1^, and RMSE = 2.646 W m^−1^ K^−1^). Therefore, composition features are used in the pre-generation BO stage.

FIG. S3 Feature-set comparison using ten-fold cross-validation. Three input representations are evaluated with the same protocol: Composition (n = 14), Elemental descriptors (n = 4), and Composition + descriptors (n = 18), where descriptors denote the four selected elemental descriptors. Bars show mean cross-validation metrics, and error bars show standard deviations across folds. The comparison supports the use of composition features in the pre-generation BO stage because the additional elemental descriptors do not improve validation performance in this setting.

## 1.9 Energy above the convex hull and thermodynamic context

To assess the thermodynamic context of the three highlighted candidates, static total-energy calculations were performed for the relaxed Ag_3_SbSe_4_, Ag_4_TiSe_4_, and Ag_4_TiTe_4_ structures using VASP with the PAW method and the PBE exchange-correlation functional. Consistent with the Materials Project calculation convention for energy cutoff and Brillouin-zone sampling, the plane-wave cutoff energy was set to 520 eV, and the k-point meshes were generated using pymatgen with a grid density of 827 divided by the number of atoms, giving a Gamma-centered 4 × 4 × 4 mesh for these candidate structures. The electronic convergence criterion was set to 4 × 10^-4^ eV. Reference competing phases in the Ag-Sb-Se, Ag-Se-Ti, and Ag-Te-Ti chemical systems were obtained from the Materials Project. The candidate entries were combined with these reference phases to construct ternary phase diagrams and evaluate the Energy above the convex hull and thermodynamic context. The resulting hull distances are 0.131 eV/atom for Ag_3_SbSe_4_, 0.049 eV/atom for Ag_4_TiSe_4_, and 0.052 eV/atom for Ag_4_TiTe_4_. These finite hull distances indicate that the three candidates should not be described as strictly thermodynamically stable; instead, the hull analysis provides thermodynamic context, while phonon spectra are used to assess dynamical stability.

FIG. S4 Ternary convex-hull diagrams for (a) Ag_3_SbSe_4_ in Ag-Sb-Se, (b) Ag_4_TiSe_4_ in Ag-Se-Ti, and (c) Ag_4_TiTe_4_ in Ag-Te-Ti. The target phases are shown as orange diamonds. Stable Materials Project entries, near-hull entries, tie lines, and decomposition phases are indicated in the legend. The diagrams provide thermodynamic context through finite energy-above-hull values and are not used as the criterion for phonon-based dynamical stability.

# 2. Comparison of calculation time for κ

To demonstrate the efficiency of our κ app, KappaP, in predicting lattice thermal conductivity, we compared its computational time with other commonly used methods. The results indicate that KappaP offers a significant speed advantage over methods based on solving the phonon Boltzmann transport equation (PBTE) using second- and third-order force constants, as well as the equilibrium molecular dynamics Green-Kubo method. For simple systems, the PBTE and Green-Kubo methods typically require several hours, while more complex systems can take days or even up to a week.

Even the semi-empirical Slack model, which relies on experimental data or theoretical calculations to determine the parameters for Eq. (3), typically takes several hours to compute. In contrast, our proposed method demonstrates a significant advantage in computational efficiency, requiring only a few seconds to predict κ and other relevant physical properties directly from a CIF file, regardless of the material.

For simple single-element systems, conventional methods may operate relatively quickly. However, the computational efficiency of KappaP becomes even more evident for complex systems, such as binary, ternary, and quaternary compounds. Traditional software like VASP encounters exponentially increasing computational challenges as the number of atoms in the primitive cell rises, particularly when calculating force constants^[16,35,36]^. In contrast, our use of the CGCNN model combined with the Slack approximation facilitates rapid pre-screening of various materials for κ, making it an effective tool for identifying materials with either high or low thermal conductivity.

# 3. Electronic transport of Ag_3_SbSe_4_

These electronic-transport calculations are used as downstream thermoelectric evaluation of candidates selected for low lattice thermal conductivity; ZT is not used in BO acquisition or LLM ranking. The electronic transport properties of Ag_3_SbSe_4_, including the Seebeck coefficient ($S$), electrical conductivity ($\sigma$), electronic thermal conductivity ($\kappa_{e}$), and power factor ($PF=S^{2}$), are presented in FIG. S5, derived from Onsager transport coefficients. To account for realistic carrier scattering mechanisms, contributions from acoustic deformation potential (ADP) scattering^[37]^, polar optical phonon (POP) scattering^[38]^, and ionized impurity (IMP) scattering^[39]^ are included.

As shown in FIG. S5(a), both n- and p-type Ag_3_SbSe_4_ exhibit large Seebeck coefficients, reaching 456 and 621 $\mu V/K$ at 300 K, respectively—comparable to the performance of SnSe^[40]^. This enhancement in $S$ originates from a high density-of-states effective mass ($m_{\mathrm{DOS}}^{*}$), which is attributed to a valence band valley degeneracy of two at the $\Gamma$ and R points, as described by the Mott relation^[41]^.

With increasing carrier concentration, both $\sigma$ and $\kappa_{e}$ increase due to the greater number of mobile charge carriers, whereas rising temperature suppresses them as a result of enhanced electron–phonon interactions, shown in FIG. S5(b-c). In contrast, the Seebeck coefficient exhibits a non-monotonic dependence on temperature at low doping levels, driven by bipolar transport effects in this narrow-gap semiconductor^[42,43]^.

As illustrated in FIG. S5(d), p-type Ag_3_SbSe_4_ consistently yields higher power factors than its n-type counterpart, reaching a peak value of 1.78 $\mathrm{mW}m^{-1}K^{-2}$ at 500 K and a carrier concentration of $4\times{10}^{20} cm^{-3}$. These results indicate that hole-doped Ag_3_SbSe_4_ merits further thermoelectric evaluation.

FIG. S5 Electronic transport parameters of Ag_3_SbSe_4_. The calculated electronic transport parameters for n-type and p-type Ag_3_SbSe_4_ at 100 K, 300 K and 500 K. (a) Electrical conductivity ($\sigma$). (b) The Seebeck coefficient ($S$). (c) The electronic thermal conductivity ($\kappa_{e}$). (d) Thermoelectric power factor ($\mathrm{PF}$).

# 4. The first-principles calculation of Ag_4_TiX_4_ (X=Se/Te)

The following transport and ZT analyses are downstream, post-screening evaluations of the low-κ candidates and are not used as BO or LLM ranking objectives. To further validate the capability of our framework in identifying materials with low lattice thermal conductivity, we investigated the thermoelectric performance of Ag_4_TiX_4_ (X = Se, Te).

FIG. S6 (a) Temperature-dependent phonon spectrum of Ag_4_TiSe_4_ from 0 K to 1100 K. b–c) Spectral and cumulative $\kappa_{p,SCPH}^{3,4ph}$ at 300 K (inset: the structure of primitive cell of g_4_TiX_4_, X = Se/Te) and thermoelectric figure of merit (*ZT*) for p-type Ag_4_TiSe_4_ as a function of carrier concentration along the a- and c-axis from 300 to 700 K. (d) Temperature-dependent phonon spectrum of Ag_4_TiTe_4_ from 0 to 1100 K. (e–f) Spectral and cumulative $\kappa_{p,SCPH}^{3,4ph}$ at 300 K and *ZT* for p-type Ag_4_TiTe_4_ as a function of carrier concentration along the a- and c-axes from 300 K to 700 K.

Ag_4_TiSe_4_ and Ag_4_TiTe_4_ both crystallize in a tetragonal structure with space group *I*-42*m* (No.121). The primitive cell parameters of Ag_4_TiSe_4_ are a = b = c = 7.3950 Å, $\alpha=\beta={130.21}^{\circ}$, $\gamma={73.08}^{\circ}$, with a unit cell volume of 230.34 Å^3^. For Ag_4_TiTe_4_, the parameters are a = b = c = 7.8893 Å, $\alpha=\beta={131.48}^{\circ}$, $\gamma={71.05}^{\circ}$, and the unit cell volume is 269.85 Å^3^. The structures form a three-dimensional framework primarily composed of Ti–X (X = Se, Te) bonds, with Ag atoms embedded within. The substitution of Se with Te leads to an increase in primitive cell volume, reflecting the larger ionic radius of Te. These structural features are expected to contribute to the low thermal conductivity, which is advantageous for thermoelectric applications.

As shown in FIG. S6(a) and FIG. S6(d), no imaginary phonon frequencies are observed under the harmonic approximation, confirming the dynamical stability of both compounds at 0 K as calculated using the PBE functional. Upon increasing the temperature up to 1100 K, both materials exhibit phonon hardening, although the frequency shifts remain small. This observation is consistent with Figs. FIG. S7 and FIG. S8, suggesting that temperature exerts a limited influence on phonon-related thermal conductivity, as further supported by the negligible variation in $\kappa_{L}$ shown in FIG. S9(a) and FIG. S9(c). Nonetheless, coherence phonon effects also make non-negligible contributions to the thermal transport of Ag_4_TiSe_4_ and Ag_4_TiTe_4_, as illustrated in FIG. S9(b) and FIG. S9(d).

FIG. S7 Projected phonon density of states (PDOS) of Ag_4_TiSe_4_ at 0 K (HA), 300 K, and 700 K.

FIG. S8 Projected phonon density of states (PDOS) of Ag_4_TiTe_4_ at 0 K (HA), 300 K, and 700 K.

FIG. S9 (a) Phonon populations’ thermal conductivity (κ_p_) as a function of the temperature of Ag_4_TiSe_4_. (b) Intrinsic lattice thermal conductivity (κ_L_) as a function of the temperature of Ag_4_TiSe_4_ . (c) Phonon populations’ thermal conductivity (κ_p_) as a function of the temperature of Ag_4_TiTe_4_. (d) Intrinsic lattice thermal conductivity (κ_L_) as a function of the temperature of Ag_4_TiTe_4_ .The solid lines represent the a-axis direction, and the dashed lines represent the c-axis direction.

To gain deeper insight, we calculate the spectral and cumulative contributions to $\kappa_{L}$, as shown in FIG. S6(b) and FIG. S6(e). For Ag_4_TiSe_4_, phonons below 50 cm^-1^ contribute 88% and 84% to $\kappa_{p}$ along the a- and c-axes, respectively. Similarly, in Ag_4_TiTe_4_, low-frequency phonons contribute 91% and 87% to $\kappa_{p}$ along the a- and c-axes. These results highlight the critical role of low-frequency vibrations—primarily associated with Ag atoms—in governing thermal transport in Ag_4_TiX_4_ (X = Se, Te), which aligns with the atomic participation ratio (APR) results for both compounds (FIG. S10), underscoring their potential as low-$\kappa_{L}$ thermoelectric materials.

FIG. S10 The color-coded atomic participation ratio (APR) of Ag_4_Ti X_4_ (X=Te, Se) projected onto the phonon bands. (a) Ag atom, (b) Ti atom and (c) Se atom of Ag_4_TiSe_4_. (d) Ag atom, (e) Ti atom and (f) Se atom of Ag_4_TiTe_4_

To evaluate the thermoelectric performance of Ag_4_TiSe_4_ and Ag_4_TiTe_4_, we calculated their electronic transport properties and determined band gaps of 0.95 eV and 0.98 eV, respectively. These band gap values fall within the range for thermoelectric materials, indicating favorable electronic structures for thermoelectric applications. The calculations reveal that p-type Ag_4_TiX_4_ (X = Se, Te) shows more favorable calculated electronic transport properties than the n-type counterparts under the tested conditions. For p-type Ag_4_TiSe_4_, the maximum power factor reaches 2.12 mW $m^{-1} K^{-2}$ at a carrier concentration of 6×10^20^ cm^−3^ and 500 K. Meanwhile, p-type Ag_4_TiTe_4_ achieves a maximum power factor of 3.43 mW $m^{-1} K^{-2}$ at 4×10^20^ cm^−3^ and 500 K, indicating favorable electronic transport behavior (FIG. S14 and FIG. S15) and supporting further thermoelectric evaluation.

As shown in FIG. S6(c) and FIG. S6(f), the maximum *ZT* values of p-type Ag_4_TiX_4_ (X = Se, Te) increase with temperature, and the optimal carrier concentrations shift toward higher values. Due to anisotropy along the a- and c-axes, Ag_4_TiSe_4_ exhibits maximum *ZT* values of 2.15 and 2.47 at 700 K along the a- and c-axes, respectively, both occurring at an optimal concentration of 2×10^20^ cm^−3^. For Ag_4_TiTe_4_, the maximum *ZT* values at 700 K are 3.15 and 3.68 along the a- and c-axes, respectively, with corresponding optimal carrier concentrations of 1×10^20^ cm^−3^ and 9×10^19^ cm^−3^. These values are higher than those of their n-type counterparts (FIG. S16).

To understand the low thermal conductivity observed in the materials Ag_4_TiX_4_ (where X = Te, Se), we analyzed the atomic participation ratio (APR) as it relates to the phonon bands. Our findings indicate that the low *κ_L_* is primarily influenced by the presence of silver (Ag) atoms, as confirmed by DFT calculations. In the low-frequency acoustic branches, the dominant contributions come from Ag, followed by tellurium (Te), while titanium (Ti) contributes minimally (FIG. S10). This suggests that Ag atoms play a significant role in reducing *κ_L_*^[35]^.

FIG. S11 (a) Specific heat capacity at constant volume (C_V_). (b) Squared phonon velocities of Ag_4_TiSe_4_ in the harmonic approximation and the anharmonic renormalization approximation at 300 K. (c) Phonon scattering rates of 3ph and 4ph for Ag_4_TiSe_4_ at 300 and 500 K. (d) Weighted phonon scattering phase space of 3ph and 4ph for Ag_4_TiSe_4_ at 300 and 700 K.

FIG. S12 (a) Specific heat capacity at constant volume (C_V_). (b) Squared phonon velocities of Ag_4_TiTe_4_ in the harmonic approximation and the anharmonic renormalization approximation at 300 K. (c) Phonon scattering rates of 3ph and 4ph for Ag_4_TiTe_4_ at 300 and 500 K. (d) Weighted phonon scattering phase space of 3ph and 4ph for Ag_4_TiTe_4_ at 300 and 500 K.

FIG. S13 Electronic band structure and electronic density of states (DOS) of (a) Ag_4_TiSe_4_ and (b) Ag_4_TiTe_4_.

FIG. S14 Electronic transport parameters of Ag_4_TiSe_4_. The calculated electronic transport parameters for n-type and p-type Ag_4_TiSe_4_ at 300, 500 and 700 K. (a) Electrical conductivity (σ). (b) The Seebeck coefficient (S). (c) The electronic thermal conductivity (κ_e_). (d) Thermoelectric power factor (PF).

FIG. S15 Electronic transport parameters of Ag_4_TiTe_4_. The calculated electronic transport parameters for n-type and p-type Ag_4_TiTe_4_ at 300, 500 and 700 K. (a) Electrical conductivity (σ). (b) The Seebeck coefficient (S). (c) The electronic thermal conductivity (κ_e_). (d) Thermoelectric power factor (PF).

FIG. S16 Calculated thermoelectric figure of merit (ZT) for (a, c) n-type and (b, d) p-type Ag_4_TiSe_4_ and Ag_4_TiTe_4_ as a function of carrier concentration along the a- and c-axes at various temperatures ranging from 300 to 700 K.

References:

[1]	T. Xie, J.C. Grossman, “Crystal Graph Convolutional Neural Networks for an Accurate and Interpretable Prediction of Material Properties,” Phys. Rev. Lett. 120, no. 14 (2018): 145301 https://doi.org/10.1103/PhysRevLett.120.145301.

[2]	S.D. Griesemer, Y. Xia, C. Wolverton, “Accelerating the prediction of stable materials with machine learning,” *Nat. Comput. Sci.* **3**, no. 11 (2023): 934–945 https://doi.org/10.1038/s43588-023-00536-w.

[3]	T. Zhu, R. He, S. Gong, T. Xie, P. Gorai, K. Nielsch, J.C. Grossman, “Charting lattice thermal conductivity for inorganic crystals and discovering rare earth chalcogenides for thermoelectrics,” *Energy Environ. Sci.* **14**, no. 6 (2021): 3559–3566 https://doi.org/10.1039/D1EE00442E.

[4]	G.A. Slack, “Nonmetallic crystals with high thermal conductivity,” *J. Phys. Chem. Solids* **34**, no. 2 (1973): 321–335 https://doi.org/10.1016/0022-3697(73)90092-9.

[5]	D.T. Morelli, G.A. Slack, 2006. “High Lattice Thermal Conductivity Solids.” in *High Thermal Conductivity Materials*. edited by ed by, S.L. Shindé, J.S. Goela New York: Springer-Verlag. pp. 37–68.

[6]	T. Jia, G. Chen, Y. Zhang, “Lattice thermal conductivity evaluated using elastic properties,” *Physical Review B* **95**, no. 15 (2017): 155206 https://doi.org/10.1103/PhysRevB.95.155206.

[7]	G. Qin, A. Huang, Y. Liu, H. Wang, Z. Qin, X. Jiang, J. Zhao, J. Hu, M. Hu, “High-throughput computational evaluation of lattice thermal conductivity using an optimized Slack model,” *Mater. Adv.* **3**, no. 17 (2022): 6826–6830 https://doi.org/10.1039/D2MA00694D.

[8]	O.L. Anderson, “A simplified method for calculating the debye temperature from elastic constants,” *J. Phys. Chem. Solids* **24**, no. 7 (1963): 909–917 https://doi.org/10.1016/0022-3697(63)90067-2.

[9]	F. Knoop, T.A.R. Purcell, M. Scheffler, C. Carbogno, “Anharmonicity measure for materials,” *Physical Review Materials* **4**, no. 8 (2020): 083809 https://doi.org/10.1103/PhysRevMaterials.4.083809.

[10]	V.N. Belomestnykh, E.P. Tesleva, “Interrelation between Anharmonicity and Lateral Strain in Quasi-Isotropic Polycrystalline Solids.,” *Tech. phys.* **49**, no. 8 (2004): 1098 https://doi.org/10.1134/1.1787679.

[11]	V.N. Belomestnykh, “The acoustical Grüneisen constants of solids,” *Tech. Phys. Lett.* **30**, no. 2 (2004): 91–93 https://doi.org/10.1134/1.1666949.

[12]	D. Chung, W. Buessem, “The voigt-reuss-hill approximation and elastic moduli of polycrystalline MgO, CaF_2_, β-ZnS, ZnSe, and CdTe,” *J. Appl. Phys.* **38**, no. 6 (1967): 2535–2540 https://doi.org/10.1063/1.1709944.

[13]	Y. Xiao, C. Chang, Y. Pei, D. Wu, K. Peng, X. Zhou, S. Gong, J. He, Y. Zhang, Z. Zeng, L.-D. Zhao, “Origin of low thermal conductivity in SnSe,” *Phys. Rev. B* **94**, no. 12 (2016): 125203 https://doi.org/10.1103/PhysRevB.94.125203.

[14]	T. Feng, X. Ruan, “Quantum mechanical prediction of four-phonon scattering rates and reduced thermal conductivity of solids,” *Physical Review B* **93**, no. 4 (2016): 045202 https://doi.org/10.1103/PhysRevB.93.045202.

[15]	F. Zhou, W. Nielson, Y. Xia, V. Ozoliņš, “Compressive sensing lattice dynamics. I. General formalism,” *Physical Review B* **100**, no. 18 (2019): 184308 https://doi.org/10.1103/PhysRevB.100.184308.

[16]	F. Zhou, W. Nielson, Y. Xia, V. Ozoliņš, “Lattice anharmonicity and thermal conductivity from compressive sensing of first-principles calculations,” *Physical Review Letters* **113**, no. 18 (2014): 185501 https://doi.org/10.1103/PhysRevLett.113.185501.

[17]	Y. Xia, V.I. Hegde, K. Pal, X. Hua, D. Gaines, S. Patel, J. He, M. Aykol, C. Wolverton, “High-throughput study of lattice thermal conductivity in binary rocksalt and zinc blende compounds including higher-order anharmonicity,” *Physical Review X* **10**, no. 4 (2020): 041029 https://doi.org/10.1103/PhysRevX.10.041029.

[18]	T. Tadano, S. Tsuneyuki, “Self-consistent phonon calculations of lattice dynamical properties in cubic SrTiO_3_ with first-principles anharmonic force constants,” *Physical Review B* **92**, no. 5 (2015): 054301 https://doi.org/10.1103/PhysRevB.92.054301.

[19]	Z. Tong, X. Yang, T. Feng, H. Bao, X. Ruan, “First-principles predictions of temperature-dependent infrared dielectric function of polar materials by including four-phonon scattering and phonon frequency shift,” *Physical Review B* **101**, no. 12 (2020): 125416 https://doi.org/10.1103/PhysRevB.101.125416.

[20]	M. Simoncelli, N. Marzari, F. Mauri, “Unified theory of thermal transport in crystals and glasses,” *Nature Physics* **15**, no. 8 (2019): 809–813 https://doi.org/10.1038/s41567-019-0520-x.

[21]	G. Kresse, J. Furthmüller, “Efficient iterative schemes for ab initio total-energy calculations using a plane-wave basis set,” *Phys. Rev. B* **54**, no. 16 (1996): 11169–11186 https://doi.org/10.1103/PhysRevB.54.11169.

[22]	P.E. Blöchl, “Projector augmented-wave method,” *Phys. Rev. B* **50**, no. 24 (1994): 17953–17979 https://doi.org/10.1103/PhysRevB.50.17953.

[23]	G. Kresse, D. Joubert, “From ultrasoft pseudopotentials to the projector augmented-wave method,” *Phys. Rev. B* **59**, no. 3 (1999): 1758–1775 https://doi.org/10.1103/PhysRevB.59.1758.

[24]	J.P. Perdew, K. Burke, M. Ernzerhof, “Generalized Gradient Approximation Made Simple,” *Phys. Rev. Lett.* **77**, no. 18 (1996): 3865–3868 https://doi.org/10.1103/PhysRevLett.77.3865.

[25]	F. Zhou, B. Sadigh, D. Åberg, Y. Xia, V. Ozoliņš, “Compressive sensing lattice dynamics. II. Efficient phonon calculations and long-range interactions,” *Phys. Rev. B* **100**, no. 18 (2019): 184309 https://doi.org/10.1103/PhysRevB.100.184309.

[26]	A. Dunn, Q. Wang, A. Ganose, D. Dopp, A. Jain, “Benchmarking materials property prediction methods: the Matbench test set and Automatminer reference algorithm,” *npj Comput. Mater.* **6**, no. 1 (2020): 138.

[27]	K. Chen, B. Song, N.K. Ravichandran, Q. Zheng, X. Chen, H. Lee, H. Sun, S. Li, G.A.G.U. Gamage, F. Tian, Z. Ding, Q. Song, A. Rai, H. Wu, P. Koirala, A.J. Schmidt, K. Watanabe, B. Lv, Z. Ren, L. Shi, D.G. Cahill, T. Taniguchi, D. Broido, G. Chen, “Ultrahigh thermal conductivity in isotope-enriched cubic boron nitride,” *Science* **367**, no. 6477 (2020): 555–559 https://doi.org/10.1126/science.aaz6149.

[28]	C. Toher, C. Oses, J.J. Plata, D. Hicks, F. Rose, O. Levy, M. de Jong, M. Asta, M. Fornari, M. Buongiorno Nardelli, S. Curtarolo, “Combining the AFLOW GIBBS and elastic libraries to efficiently and robustly screen thermomechanical properties of solids,” *Phys. Rev. Mater.* **1**, no. 1 (2017): 015401 https://doi.org/10.1103/PhysRevMaterials.1.015401.

[29]	X. Wang, G. Shu, G. Zhu, J.-S. Wang, J. Sun, X. Ding, B. Li, Z. Gao, “An interpretable formula for lattice thermal conductivity of crystals,” *Mater. Today Phys.* **48** (2024): 101549.

[30]	J. Hu, Y. Zuo, Y. Hao, G. Shu, Y. Wang, M. Feng, X. Li, X. Wang, J. Sun, X. Ding, Gao Zhibin, G. Zhu, B. Li, “Prediction of lattice thermal conductivity with two-stage interpretable machine learning,” *Chin. Phys. B* **32**, no. 4 (2023): 046301 https://doi.org/10.1088/1674-1056/acbaf4.

[31]	A. Merchant, S. Batzner, S.S. Schoenholz, M. Aykol, G. Cheon, E.D. Cubuk, “Scaling deep learning for materials discovery,” *Nature* **624**, no. 7990 (2023): 80–85 https://doi.org/10.1038/s41586-023-06735-9.

[32]	M. Feng, X. Wang, G. Zhu, C. He, J. Sun, X. Ding, J. Shiomi, Y. Xia, B. Li, Z. Gao, “The relation between the atomic mass ratio and quartic anharmonicity in alkali metal hydrides,” *Mater. Today Phys.* **44** (2024): 101423.

[33]	X. Wang, M. Feng, Y. Xia, J. Sun, X. Ding, B. Li, Z. Gao, “Revisiting lattice thermal conductivity of CsCl: The crucial role of quartic anharmonicity,” *Appl. Phys. Lett.* **124**, no. 17 (2024) https://doi.org/10.1063/5.0201393.

[34]	Y. Wang, Z. Gao, X. Wang, J. Sun, M. Feng, Y. Hao, X. Li, Y. Zhao, X. Ding, “Anomalous thermal conductivity in 2D silica nanocages of immobilizing noble gas atom,” *Appl. Phys. Lett.* **124**, no. 12 (2024) https://doi.org/10.1063/5.0200462.

[35]	X. Wang, M. Li, M. Feng, X. Li, Y. Hao, W. Shi, J. He, X. Ding, Z. Gao, “Bonding hierarchy and coordination interaction leading to high thermoelectricity in wide bandgap TlAgI_2_,” *Phys. Rev. Mater.* **8**, no. 9 (2024): 094601 https://doi.org/10.1103/PhysRevMaterials.8.094601.

[36]	Y. Hao, Y. Zuo, J. Zheng, W. Hou, H. Gu, X. Wang, X. Li, J. Sun, X. Ding, Z. Gao, “Machine learning for predicting ultralow thermal conductivity and high ZT in complex thermoelectric materials,” *ACS Appl. Mater. Interfaces* **16**, no. 36 (2024): 47866–47878 https://doi.org/10.1021/acsami.4c09043.

[37]	J. Bardeen, W. Shockley, “Deformation potentials and mobilities in non-polar crystals,” *Physical Review* **80**, no. 1 (1950): 72–80 https://doi.org/10.1103/PhysRev.80.72.

[38]	H. Fröhlich, “Electrons in lattice fields,” *Advances in Physics* **3**, no. 11 (1954): 325–361 https://doi.org/10.1080/00018735400101213.

[39]	A.M. Ganose, J. Park, A. Faghaninia, R. Woods-Robinson, K.A. Persson, A. Jain, “Efficient calculation of carrier scattering rates from first principles,” *Nature Communications* **12**, no. 1 (2021): 2222 https://doi.org/10.1038/s41467-021-22440-5.

[40]	L.-D. Zhao, S.-H. Lo, Y. Zhang, H. Sun, G. Tan, C. Uher, C. Wolverton, V.P. Dravid, M.G. Kanatzidis, “Ultralow thermal conductivity and high thermoelectric figure of merit in SnSe crystals,” *Nature* **508**, no. 7496 (2014): 373–377 https://doi.org/10.1038/nature13184.

[41]	G.D. Mahan, J.O. Sofo, “The best thermoelectric.,” *Proceedings of the National Academy of Sciences* **93**, no. 15 (1996): 7436–7439 https://doi.org/10.1073/pnas.93.15.7436.

[42]	J.J. Gong, A.J. Hong, J. Shuai, L. Li, Z.B. Yan, Z.F. Ren, J.-M. Liu, “Investigation of the bipolar effect in the thermoelectric material CaMg_2_ Bi_2_ using a first-principles study,” *Physical Chemistry Chemical Physics* **18**, no. 24 (2016): 16566–16574 https://doi.org/10.1039/C6CP02057G.

[43]	R. Zhu, Z. Gao, Q. Liang, J. Hu, J.-S. Wang, C.-W. Qiu, A.T.S. Wee, “Observation of anisotropic magnetoresistance in layered nonmagnetic semiconducting PdSe_2_,” *ACS Applied Materials & Interfaces* **13**, no. 31 (2021): 37527–37534 https://doi.org/10.1021/acsami.1c10500.
